# Targeted eicosanoids profiling reveals a prostaglandin reprogramming in breast Cancer by microRNA-155

**DOI:** 10.1186/s13046-021-01839-4

**Published:** 2021-01-25

**Authors:** Sinae Kim, Eun Sung Lee, Eun ji Lee, Jae Yun Jung, Sae Byul Lee, Hee Jin Lee, Jisun Kim, Hee Jeong Kim, Jong Won Lee, Byung Ho Son, Gyungyub Gong, Sei-Hyun Ahn, Suhwan Chang

**Affiliations:** 1 Department of Biomedical Sciences, Asan Medical Center, University of Ulsan College of Medicine, Seoul, 05505 South Korea; 2 Division of Breast Surgery, Department of Surgery, Asan Medical Center, University of Ulsan College of Medicine, Seoul, 05505 South Korea; 3 Department of Pathology, Asan Medical Center, University of Ulsan College of Medicine, Seoul, 05505 South Korea

**Keywords:** Microrna-155, Prostaglandin E2, Prostaglandin D2, PTGES1, PTGES2, Myc, KLF4, TNBC

## Abstract

**Abstract:**

**Background:**

Prostaglandin is one of the key metabolites for inflammation-related carcinogenesis. Despite the microRNA-155 is implicated in various types of cancers, it’s function in prostaglandin metabolism is largely unknown.

**Methods:**

A targeted profiling of eicosanoids including prostaglandin, leukotriene and thromboxanes was performed in miR-155 deficient breast tumors and cancer cells. The molecular mechanism of miR-155-mediated prostaglandin reprogramming was investigated in primary and cancer cell lines, by analyzing key enzymes responsible for the prostaglandin production.

**Results:**

We found miR-155-deficient breast tumors, plasma of tumor-bearing mouse and cancer cells show altered prostaglandin level, especially for the prostaglandin E2 (PGE2) and prostaglandin D2 (PGD2). Subsequent analysis in primary cancer cells, 20 triple-negative breast cancer (TNBC) specimens and breast cancer cell lines with miR-155 knockdown consistently showed a positive correlation between miR-155 level and PGE2/PGD2 ratio. Mechanistically, we reveal the miR-155 reprograms the prostaglandin metabolism by up-regulating PGE2-producing enzymes PTGES/PTGES2 while down-regulating PGD2-producing enzyme PTGDS. Further, we show the up-regulation of *PTGES2* is driven by miR-155-cMYC axis, whereas *PTGES* is transactivated by miR-155-KLF4. Thus, miR-155 hires dual-regulatory mode for the metabolic enzyme expression to reprogram the PGE2/PGD2 balance. Lastly, we show the miR-155-driven cellular proliferation is restored by the siRNA of PTGES1/2, of which expression also significantly correlates with breast cancer patients’ survival.

**Conclusions:**

Considering clinical trials targeting PGE2 production largely have focused on the inhibition of Cox1 or Cox2 that showed cardiac toxicity, our data suggest an alternative way for suppressing PGE2 production via the inhibition of miR-155. As the antagomiR of miR-155 (MRG-106) underwent a phase-1 clinical trial, its effect should be considered and analyzed in prostaglandin metabolism in tumor.

**Supplementary Information:**

The online version contains supplementary material available at 10.1186/s13046-021-01839-4.

## Background

microRNA-155 is encoded by a non-coding, BIC (B-cell lymphoma Insertion Cluster) gene, discovered as an oncogene that is activated by retroviral insertion [1]. As miR-155 is the only microRNA produced from the BIC, the oncogenic function of the BIC gene is driven by miR-155. In addition to its function in lymphoid cell transformation [2], miR-155 has shown oncogenicity in solid tumors including lung, colon, pancreatic, and breast cancer [3]. Based on the nature of incomplete miRNA-target base-paring, the number of miR-155 targets was estimated to be more than 200, which was verified by the Ago-CLIP results [4]. Because of the large number of targets, it is difficult to dissect the molecular mechanism of the miR-155-induced oncogenicity. In cancer cells, miR-155 affects multiple biochemical, physiological aspects of cancer such as proliferation, migration, drug resistance, autophagy, and immune evasion [5]. Notably, several recent reports revealed the function of miR-155 in cancer metabolism including glucose [6], thiamine [7] and estrogen metabolism [8], thereby suggested the molecular function of miR-155 on deregulated metabolic control in cancer cells.

Eicosanoids are 20-carbon bioactive lipids generated from fatty acids, with diverse biological functions related to cancer, including cell growth, migration, and angiogenesis [9] . The biogenesis of eicosanoids starts from arachidonic acid, generated from phospholipid by PLA2; subsequently, it is metabolized into leukotrienes and prostanoids [10]. Prostaglandin is a set of lipid metabolite of prostanoids with diverse physiological functions, which are derived from arachidonic acids and have 20 carbons with a 5-carbon ring. For each of the prostaglandin, there is a synthase responsible for its biogenesis [11]. Among the prostaglandins, PGE2 is well-known for its oncogenic function via EP receptors which in turn activates the PKA-CREB pathway, Ras-Raf-MAPK, or PI3K-AKT pathway [12, 13]. In contrast, PGD2, an antitumor prostaglandin, reduces the expression of cMYC and Cyclin D1 and increases apoptosis [14]. Multiple studies have shown that microRNAs control the synthesis of eicosanoids: for example, Cox2, a key enzyme for the synthesis of PGH2 from arachidonic acid, is regulated by miR-101a, miR-146a, miR-16, and miR-26b [15–18]. Similarly, 5-LO and FLAP, which are responsible for 5-HPETE biogenesis, are controlled by miR-219 and miR-135a, respectively. Despite these results, it is unclear how miR-155 contributes to eicosanoid homeostasis in cancer. In this study, we performed eicosanoids profiling in miR-155-deficient breast cancer cells and investigated the oncogenic role of miR-155 on prostaglandin biogenesis and its clinical impact on breast cancer.

## Methods

### Human TNBC primary culture and sample analysis

Human cancer samples were obtained from patients diagnosed with triple negative breast cancer (TNBC) (Asan Medical Center, IRB No.2013–0939). We used 71 formalin-fixed, paraffin-embedded (FFPE) tissues of TNBC samples as previously described [19]. Briefly, total miRNAs were extracted from 8-μm thick FFPE tissues by using a miRNeasy FFPE kit (Qiagen). Among them, we selected 15 samples from each group, based on the frozen tissue availability at our Bio Resource Center. Human primary breast cancer cells were obtained from patients with triple-negative breast cancer (TNBC) as previously described [19]. Depending on the level of miR-155, six of the primary cells were grouped as patient-derived cells (PDCs) H1-H3 and L1-L3. The human primary cells were cultured in RPMI1640 medium with 10% FBS, hEGF (200 ng/ml), hydrocortisol (10 mg/ml), transferrin (10 mg/ml), and 1% penicillin/streptomycin.

### Animal study

Protocols for animal experiment was reviewed and approved by the Institutional Animal Care and Use Committees (IACUC) of Asan Institute for Life Sciences (AILS, Project Number: 2018–12-239). All mice were maintained in the specific pathogen–free (SPF) facility of the Laboratory of Animal Research at the Asan Institute for Life Sciences (Seoul, South Korea).

**Fig. 1:**
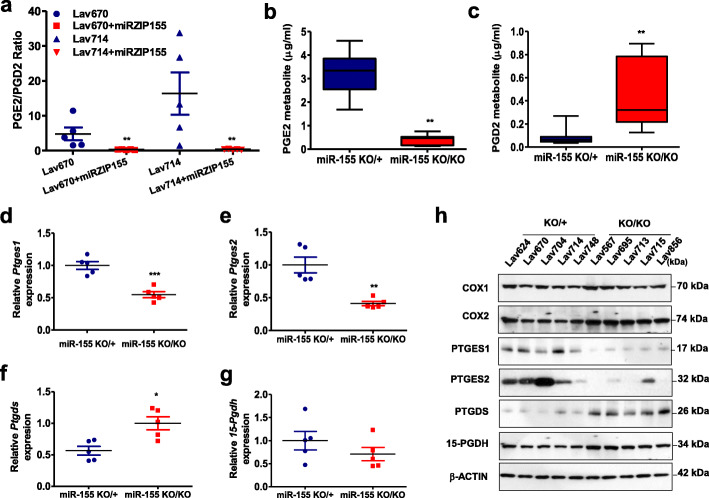
miR-155 depletion in murine mammary tumors results in disrupted PGE2/PGD2 balance and abnormal expressions of related metabolic enzymes. **a** PGE2/PGD2 measurement from two sets of mammary tumors with stable knockdown of miR-155 (marked as Lav670 + miRZIP155 and Lav714 + miRZIP155), showing significantly reduced PGE2/PGD2 ratio (*n* = 20). **b-c** Two graphs showing PGE2(**b**) or PGD2(**c**) level, from the plasma samples of miR-155 ^KO/KO^ or miR-155^KO/+^ mouse with mammary tumors (*n* = 10). **d-g** Relative RNA expression of *Ptges1*(**d**), *Ptges2*(**e**), *Ptgds*(**f**) and *15-Pgdh*(**g**) analyzed in miR-155 ^KO/KO^ or miR-155^KO/+^ mammary tumors (*n* = 10). **h** Western blot analysis of key metabolic enzymes of PGE2/PGD2 production in miR-155 ^KO/KO^ and miR-155^KO/+^ mammary tumors (*n* = 10). COX1, COX2, PTGES1, PTGES2, PTGDS, and 15-PGDH expressions are shown. β-actin was probed to ensure equal loading

The mammary gland mouse model in miR-155 deficient background was generated by crossing a Brca1^cko/cko^;Trp53^cko/cko^; K14-Cre (mammary epithelial cell-specific, conditional knockout model) mouse with miR-155^ko/ko^ mouse [20]. We obtained primary tumor cells (labelled with Lav) and plasma samples from mouse with tumors spontaneously developed in 6–9 months after birth. Ten mice for each group of miR-155 ^ko/+^ or miR-155^ko/ko^ were used in the study. For allograft experiment (in Fig. 1a), 5 mice for each group were analyzed.

### Cell culture and transfection

The miR-155-deficient breast cancer mouse model was generated as previously described. miR-155 heterozygous (miR-155 ^KO/+^) or knockout (miR-155 ^KO/KO^) primary cancer cells were isolated from tumor tissues using a modified MEC (mammary epithelial cell) isolation procedure as previously described [21]. The mouse primary cancer cells, MCF7, Hs-578 T, MDA-MB-436 and 293TN (for virus packaging; System Biosciences, SBI, Mountain View, CA, USA) were maintained in DMEM containing 10% FBS and 1% antibiotics. All plasmids and siRNAs were transfected into different cells using lipofectamine 2000 (Invitrogen) according to the manufacturer’s instructions. Small interfering RNAs (siRNAs) against human PTGES1, PTGES2, PTGDS and FOXO3a were purchased from Genolution Inc. (Seoul, Republic of Korea). The siRNA sequences are listed in Supplementary Table 8. The cells were transfected with either 200 nmol/L of target siRNAs or scramble siRNA using lipofectamine 2000 (Invitrogen). For the overexpression of PTGDS, pCNS-PTGDS vector was purchased from Korea Human Gene Bank (KHGB, Daejeon, Korea). The PTGDS fragment was introduced into pcDNA3 vector via EcoRI and NotI sites. The pcDNA3-PTGDS were transected using lipofectamine 2000 (Invitrogen) and used for further experiments of western blot and cell viability assay.

### Enzyme-linked immunosorbent assay (ELISA)

Blood samples were obtained from patients with triple-negative breast cancer (TNBC) (Asan Medical Center, IRB No.2013–0939). The plasma concentrations of PGE2 and PGD2 were determined using ELISA kits from ENZO Life Sciences (Farmingdale, New York, NY, USA) and CUSABIO Biotechnology (Wu Han, China), respectively. An aliquot of 10 μl of serum added to 90 μl of diluent (0.1% bovine serum albumin, 0.05% Tween 20, 10 mM Tris and 150 mM sodium chloride, pH 7.3) was used for analysis.

### LC-MS/MS analysis

A liquid chromatography-tandem mass spectrometry (LC-MS/MS) system was used to measure metabolites according to a previously described method [7].

### Quantitative RT-PCR (qRT-PCR)

Total RNA extraction was performed using TRizol (Invitrogen) according to the manufacturer’s instruction. The primer sequences are shown in Supplementary Table 8. The mRNA expression levels of major eicosanoids related genes (*PTGES1, PTGES2, PTGDS, 15-PGDH*) and *KLF4* genes were measured by real-time RT-PCR according to the manufacturer’s protocol. Relative expression value was normalized to *RPL13a* and calculated by using the 2-(ΔΔCt) method. Quantitative measurement of miR-155 was performed according to the previously described method [21]. The expression was normalized with small nuclear RNA U6 (RNU6).

### Antibody and Western blot analysis

Preparation of total cell lysates and Western blot analysis were performed as previously described. A total of 10–50 μg of protein was used per lane. The blot was probed with anti-COX1 (1:1000, Cell Signaling Technology, Danvers, MA, USA), anti-COX2 (1:1000, Cell Signaling), anti-PTGES1 (1:500; Santa Cruz Biotechnology, Santa Cruz, CA, USA), anti-PTGES2 (1:1000, Santa Cruz Biotechnology), PTGES2 (1:1000, Santa Cruz Biotechnology), anti-PTGDS (1:1000, Santa Cruz Biotechnology), anti-15-PGDH (1:1000, Santa Cruz Biotechnology), anti-KLF4 (1:1000, Cell Signaling), anti-cMYC (1:1000, Cell Signaling), FOXO3a (1:1000, Cell Signaling) and anti-β-actin (11,000, Santa Cruz Biotechnology) antibodies. The relative densities of bands were analyzed with the NIH Image J 1.47v software.

### miR-155 knockdown or overexpression

Lentiviral vector system from SBI (Mountain View, CA, USA) was used for miR-155 inhibition (miRZIP155). The lentiviral production was performed according to the manufacturer’s instructions. Control or miRZIP155-containing lentivirus particles were infected into MDA-MB-436 and Hs-578 T. After 24 h, cells were selected by Puromycin (1 mg/ml; Sigma) for 7 days. For miR-155 overexpression, a lentivirus was made using a miR-155 overexpression vector (miRH155 or control) according to the same procedure as miRZIP155 virus. MCF7 and human primary breast cancer cells (PDCL1-L3) were infected with miRH155 lentiviral vectors for 24 h.

### Chromatin immunoprecipitation (ChIP) assay

ChIP was performed according to a protocol (http://cshprotocols.cshlp.org/content/2009/9/pdbprot5279.full) with minor modifications. Briefly, MDA-MB-436 cells infected with control or miRZIP155 lentiviral vectors were cross-linked with 1% formaldehyde for 10 min at RT. The cells were then collected, lysed, sonicated, and incubated for overnight with a C/EBP-β antibody. PCR was used to detect ChIP signal with the primers listed in Supplementary Table S8.

### Reporter construction and luciferase assay

For reporter construction, the promoter regions of PTGES1 and PTGES2 were amplified (primer sequences in Appendix Table S8) and introduced into the pGL3 Enhancer vector (Promega) via *Spe*I and *Hin*dIII sites. MDA-MB-436 and Hs-578 T cells (infected with control or miRZIP155 lentiviral vectors) were transfected with reporter vectors (pGL3-PTGES1 or pGL3-PTGES2 or Empty vector) with or without overexpression vector (pcDNA3-cMYC or pEIGW-human KLF4, gift from Dr. Han Seok Choi, University of Ulsan College of Medicine, Seoul, Republic of Korea). After 48 h, luciferase activity was measured using Dual-Luciferase Reporter Assay System (Promega Corp, WI, USA) and Victor Luminometer (Perkin-Elmer, MA, USA).

### alamarBlue® cell viability assay

To test whether the inhibition of *PTGES1* or *PTGES2* using siRNAs can reverse the increase in the growth of cancer cell triggered by miR-155 or PGE2, we performed two methods using lentiviral vector encoding miR-155 (miRH155) and PGE2. The cells infected with miRH155 were transfected with siPTGES1 or siPTGES2 in a 96 well plate. After 48 h, 1/10th volume of alamarBlue® reagent (Invitrogen) was directly added to the culture media. The cells were incubated for additional 4 h to assay viability, which was detected by fluorescence measurements using a microplate fluorescence spectrophotometer (GenTeks Biosciences, Inc., San-Chong, Taipei). For induction of proliferation by PGE2, the cells were pre-treated with 100 mM PGE2 for 4 days and transfected with siPTGES1 or siPTGES2 plus PGE2. The procedure is same as mentioned above.

### Statistical analysis

All data are reported as mean ± standard error. Comparisons between two groups were performed using the Student’s *t*-test. For multiple groups, the data were analyzed by ANOVA test. *P* values < 0.05, < 0.01 and < 0.001 were marked as *, ** and *** respectively, to indicate statistical significance.

## Results

### Eicosanoids profiling of miR-155-deficient mammary tumors revealed disrupted prostaglandin balance and related synthetase expression in the absence of miR-155

We have previously reported the role of miR-155 on the MDSC recruitment regulation [20] and glucose metabolism [19] by using a breast cancer mouse model with miR-155 deficiency. On the other hand, another group showed that miR-155 enhances cyclooxigenase-2 (COX-2) expression and PGE2 secretion in asthmatic and non-asthmatic hASMC [22], raising the possibility that miR-155 has a role on prostaglandin expression in tumors as well. Therefore, we conducted a targeted profiling of eicosanoids, of which prostaglandins are included as a subfamily. We first analyzed two paired-sets of miR-155 depleted, mammary tumor samples from allograft model [20] (Fig. 1a, marked as miRZIP155). Our targeted LC-MS/MS analysis detected 30 isocyanides, including leukotrienes, thromboxanes, and prostaglandins (Supplementary Table 1 for raw data). Among these, we found evident decrease in PGE2/PGD2 ratio upon the depletion of miR-155, in both sets of mammary tumors (Fig. 1a). We further examined if miR-155 status correlates with PGE2/PGD2 expression, using the miR-155 ^KO/+^ and miR-155 ^KO/KO^ breast tumor model [23]. The results in Fig. 1b and c indicate the plasma from miR-155 ^KO/KO^ tumor model contains less PGE2 and more PGD2 than miR-155 ^KO/+^ tumor model (Supplementary Table 2 for raw data), supporting the result in Fig. 1a.

PGE2 is known as an inflammatory and oncogenic metabolite in multiple tumor types [24], whereas PGD2 was speculated tumor inhibitory in gastric cancer [25]. Therefore, the increase ratio of PGE2/PGD2 in miR-155-positive tumor and plasma suggested a signature of oncogenic prostaglandin shift. We thus investigated target genes of miR-155, possibly involved in the prostaglandin shift. There are three enzymes that control the level of PGE2: PTGES1 (Prostaglandin E2 Synthase 1), PTGES2, and 15-PGDH (15-Hydroxyprostaglandin dehydrogenase). The former two enzymes synthesize PGE2 from PGH2 [26], and the 15-PGDH inactivates PGE2 by oxidizing the 15-hydroxyl group [27]. Consistent with the positive correlation between microRNA-155 and the PGE2 level, we observed that miR-155-deficient tumors had decreased RNA levels of PTGES1 or PTGES2 (Fig. 1d and e) and increased level of PTGDS (Fig. 1f), while 15-PGDH level was not significantly changed (Fig. 1g). Western blot analysis in Fig. 1h confirmed the RT-PCR data, showing down-regulated PTGES1/2 and up-regulated PTGDS in miR-155-deficient tumors. These data collectively suggest a strong interaction between miR-155 and prostaglandin expression, which is likely mediated by the regulation of enzymes responsible for the synthesis of PGE2/PGD2.

### Eicosanoids profiling in TNBC with known miR-155 status confirms the miR-155 correlates with PGE2/PGD2 balance via synthetase regulation

**Fig. 2:**
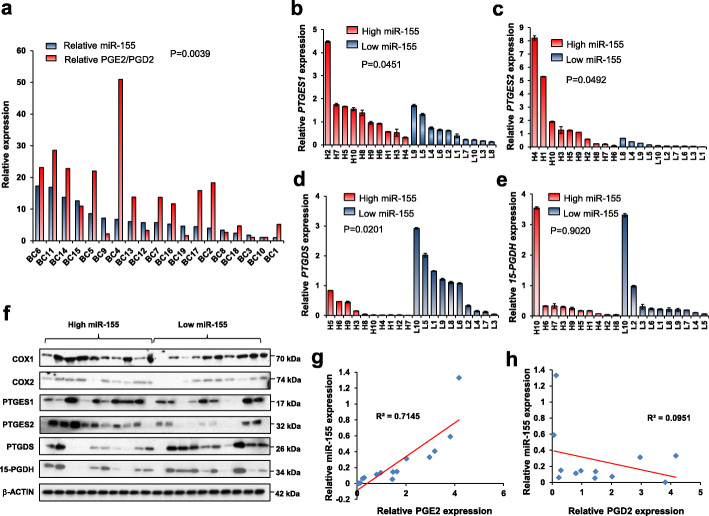
Eicosanoids profiling in triple negative breast cancer (TNBC) reveals miR-155 positively correlates with PGE2/PGD2 ratio and related metabolic enzyme expression. **a** Expression-correlation analysis of miR-155 (in blue) and PGE2/PGD2 ratio (in red) measured in human TNBC specimens (*n* = 20). **b-e** Relative RNA expression of *PTGES1*(**b**), *PTGES2*(**c**), *PTGDS*(**d**) and *15-PGDH*(**e**) in miR-155-high (H1-H10, in red) and miR-155-low (L1-L10, in blue) TNBC specimens (*n* = 20). **f** Western blot results of key metabolic enzymes and suggested novel regulators for PGE2/PGD2 metabolism in the miR-155-high and -low TNBC specimens. Results of COX1, COX2, PTGES1, PTGES2, PTGDS and 15-PGDH are shown. **g**, **h** Correlation analysis between miR-155 and PGE2(**g**) or PGD2(**h**) level in the plasma samples of TNBC patients

Our previous analysis showed the miR-155 is highly expressed in human triple negative breast cancer (TNBC) [21]. Hence, we questioned if the correlation between miR-155 and prostaglandin balance observed in knockout mouse model is also valid in TNBC specimens. First, we measured the level of PGE2/PGD2 in 20 TNBCs with various expression levels of miR-155 and observed a significant positive correlation between PGE2/PGD2 ratio and miR-155 (*p* = 0.045; Fig. 2a, raw data in Supplementary Table 3). Moreover, when we divided the 20 tumors into miR-155-high and miR-155-low groups, we observed that miR-155-high group had higher PTGES1, PTGES2 and lower PTGDS expressions, than the miR-155-low group (Fig. 2b-d). In contrast, there was no significant difference in the level of 15-PGDH (Fig. 2e). The correlations in RNA level were further validated by Western blot analysis for the three enzymes, PTGES1, PTGES2 and PTGDS (Fig. 2f). Moreover, a correlation analysis between miR-155 expression and PGE2/PGD2 level in the plasma of TNBC patients also supported our results (Fig. 2g and h, patient information in Supplementary Table 4 and raw data in Supplementary Table 5). These data collectively demonstrate a correlation between miR-155 and PGE2/PGD2 balance is conserved from mouse to human TNBC.

### microRNA-155 shifts PGE2/PGD2 balance in human breast cancer cells via the regulation of PTGES/PTGES2/PTGDS expression

**Fig. 3:**
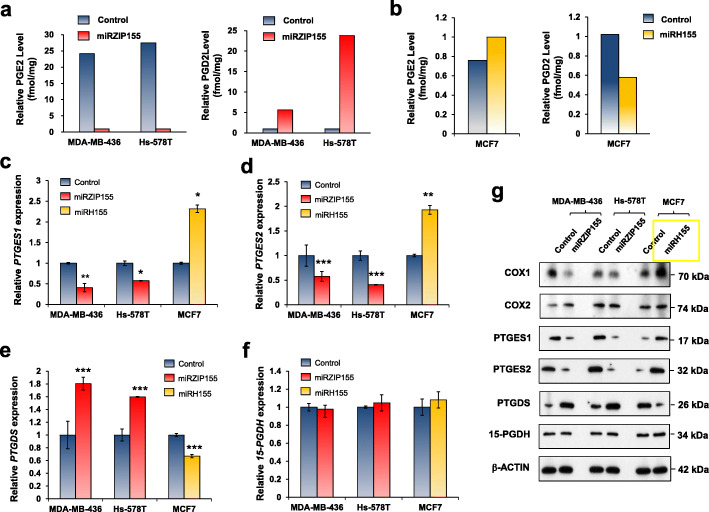
Modulation of miR-155 reprograms PGE2 and PGD2 production in human breast cancer cells, by the regulation of three prostaglandin synthases. **a** Relative PGE2 (left) and PGD2 (right) levels in MDA-MB-436 or Hs-578 T cells in control (in blue) or in miR-155-depleted cells (miRZIP155 in red). **b** The overexpression of miR-155 (miRH, in yellow) drives increased PGE2 (left) and decreased PGD2 (right) expression in MCF7 cells. **c-f** Relative RNA expression of *Ptges1*(**c**), *Ptges2*(**d**), *Ptgds*(**e**) and *15-Pgdh*(**f**) in MDA-MB-436, Hs-578 T, and MCF7 cells after knockdown (miRZIP155, in red) or overexpression (miRH155, in yellow) of miR-155. **g** Western blot analysis of key metabolic enzymes for PGE2/PGD2 homeostasis, after knockdown (miRZIP155) or overexpression (miRH155) of miR-155 in three breast cancer cell lines

Based on the results obtained from the miR-155-deficient mouse model, we further examined the role of miR-155 on prostaglandin metabolism in human breast cancer cells. Our previous study revealed miR-155 is up-regulated in MDA-MB-436 and Hs-578 T cells while down-regulated in MCF7 [19]. Thus, we depleted miR-155 in MDA-MB-436 and Hs-578 T cells while overexpressed it in MCF7, by lentivirus-mediated antagomir/mimic of miR-155. The results of which are shown in Supplementary Fig. 1a and 1b. Consistent with the data from miR-155 deficient mouse model, we observed decreased PGE2 and increased PGD2 levels in MDA-MB-436 and Hs-578 T upon miR-155 depletion (Fig. 3a, Supplementary Table 6 for raw data). Conversely, we observed increased PGE2 and decreased PGD2 in MCF7 cells, upon miR-155 overexpression (Fig. 3b). We further observed reduced PTGES1/2 and increased PTGDS expression levels in miR-155-depleted cells (Fig. 3c-e, red bars). In contrast, the overexpression of miR-155 in MCF7 cells resulted in increased PTGES1/2 and reduced PTGDS expression levels (Fig. 3c-e, yellow bars). We found no significant changes in 15-PDGH in all three cells tested (Fig. 3f). These results were validated in the protein level by Western blot (Fig. 3g).

We further confirmed our findings in patient derived breast cancer cells (PDCs). The overexpression of miR-155 in three independent miR-155-low PDCs (PDCL1–3 in Supplementary Fig. S1c) replicated the results obtained from the miR-155-overexpressed MCF7 cells (Supplementary Fig. S2a-S2b, raw data in Supplementary Table 7). Importantly, exogenous miR-155 expression (marked as miRH155) in the miR-155-low PDCs restored the expression levels of PGE2/PGD2, PTGES1/2 and PTGDS to the level of miR-155-high PDCs (yellow bars in Supplementary Fig. S3a-S3c), and these results were validated by Western blot analysis (Supplementary Fig. S3e). Two of the miR-155-overexpressed PDCL (PDCL1 and 3) showed slightly increased RNA levels of 15-PGDH (Supplementary Fig. S3d) but no significant changes in the protein level (Supplementary Fig. S3e).

### PTGES2 expression is positively regulated by the miR-155-Myc axis

miR-155 regulates the expression of multiple genes including C/EBP-β, cMYC, FOXO3a, thiamine synthase, and GLUT1 via the direct regulation on 3’UTR or indirect regulation via the transcriptional regulator of the target genes [7, 19, 23]. As we observed that the alteration of miR-155 level changes the expression levels of PTGES1/2 and PTGDS, we investigated if such regulation is carried out in a direct manner or via other transcription factors. Considering that miRNA mostly represses gene expression, positive correlation between PTGES1 or PTGES2 and miR-155 suggested an indirect regulation. Indeed, UTR analysis of the two genes in TargetScan revealed no putative miR-155 binding sites (Supplementary Fig. S4a and S4b).

**Fig. 4:**
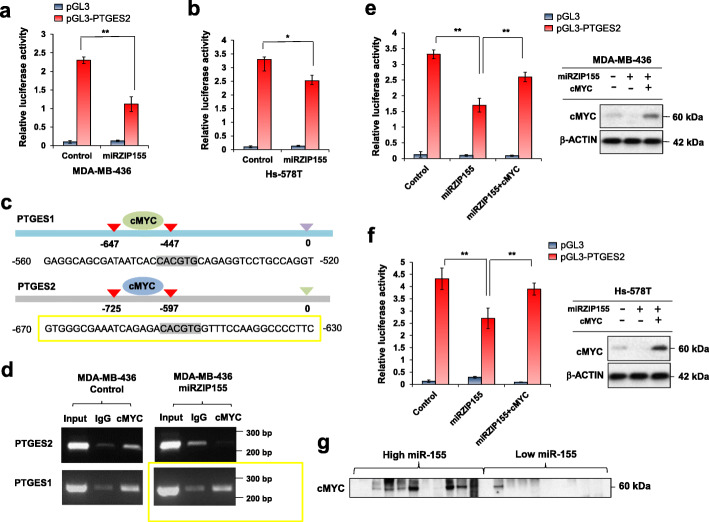
cMYC mediates the miR-155-driven PTGES2 transactivation. **a**, **b** Promoter activity of PTGES2 gene in MDA-MB-436 (**a**) and Hs-578 T cells (**b**) with (miRZIP155) or without (control) miR-155 knockdown. PGL3 empty vector was used as negative control. **c** Schematic diagram showing the cMYC-binding site (E-box, marked in grey) on PTGES1 or PTGES2 gene promoter. Numbers indicate nucleotide positions counted from the transcription start site. **d** Chromatin immunoprecipitation result of cMYC in MDA-MB-436 control and knockdown (miRZIP155) cells. Promoters of PTGES1 and PTGES2 are tested. IgG was used as negative control. **e**, **f** Promoter activity of PTGES2 gene in MDA-MB-436(**e**) and Hs-578 T cells (**f**) with miR-155 knockdown (miRZIP), in combination with cMYC reconstitution (miRZIP155 + cMYC). Data on the right panels confirms cMYC protein overexpressed in the reporter assay.**g** Western blot results of cMYC in the miR-155-high and -low TNBC specimens (*n* = 20)

Therefore, we hypothesized a presence of mediators (possibly transcription factor) which are regulated by miR-155, responsible for the control of PTGES1/2 gene expression. Figure 4a and b show that miR-155 knockdown reduces the promoter activity of PTGES2, suggesting that miR-155 regulates PTGES2 expression via a transcription factor acting on its promoter. Among the candidates, we focused on cMYC as a strong oncogene [28] and our previous study identified cMYC as a gene with a positive correlation with miR-155 [19]. The ENCODE data analysis revealed cMYC ChIP signals on both PTGES1 and 2 (Supplementary Fig. S5a and S5b), which was confirmed in MDA-MB-436 cells (Fig. 4c and d). However, we found that the binding of cMYC on PTGES1 promoter was unaltered by the miR-155 knockdown, suggesting an alternative mechanism for PTGES1. Further analysis in MDA-MB-436 and Hs578T breast cancer cells showed that the expression of cMYC restores the miR-155 knockdown-induced reduction in PTGES2 promoter activity (Fig. 4e and f). We also confirmed an elevated Myc expression in miR-155 high TNBC samples analyzed in Fig. 2 (Fig. 4g), supporting our cell line data. These results demonstrate that cMYC contributes, at least in part, to the miR-155-mediated regulation of PTGES2 expression.

### PTGES1 expression is positively regulated by the miR-155-KLF4 axis

**Fig. 5:**
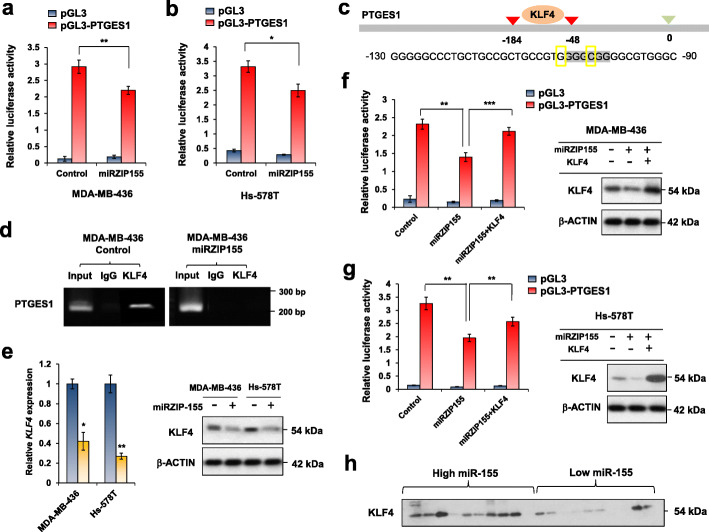
KLF4 mediates the miR-155-driven PTGES1 transactivation. **a**, **b** Promoter activity of PTGES1 gene in MDA-MB-436(**a**) and Hs-578 T(**b**) cells with control (in blue) or miR-155 knockdown (in red). **c** Schematic diagram showing the KLF4-binding site on the PTGES1 promoter. Numbers indicate nucleotide positions counted from the transcription start site. **d** Chromatin immunoprecipitation result of KLF4 in MDA-MB-436 control or in miR-155 knockdown (miRZIP155) cells. **e** Relative RNA expression of KLF4 in MDA-MB-436 and Hs-578 T cells. Control (in blue) or miR-155 knockdown cells (miRZIP155, in yellow) were tested. Data on the right panel shows KLF4 protein level. **f**, **g** PTGES1 promoter reporter assay with KLF4 reconstitution, in MDA-MB-436(**f**) or in Hs-578 T(**g**) cells. The panels on right show the KLF4 protein level overexpressed. **h** Western blot results of KLF4 in the miR-155-high and -low TNBC specimens (*n* = 20)

The ChIP data for PTGES1 in Fig. 4d indicated cMYC binds to PTGES1 promoter but independently to miR-155 status. Promoter assay analysis for PTGES1 showed that miR-155 knockdown decreases the promoter activity of PTGES1 (Fig. 5a and b), implying a presence of transcription activator, suppressed by miR-155. Therefore, we examined if Foxo3a, a well-known target of miR-155 [19], can regulates PTGES1/2. The results in Supplementary Figure S6a shows the knockdown of Foxo3a did not affect PTGES2 level, although the PTGES1 expression was increased marginally. This data suggest there might be more important mediator(s) for the miR-155-driven PTGES regulation. Among the other candidates, KLF4 drew our attention as a recent report showed a positive correlation between KLF4 and PGE2 in M2 macrophages [29]. The prediction of transcription factor interaction on PTGES1 promoter showed a KLF4 binding site 184 nt upstream of TSS (Fig. 5c). We confirmed KLF4 binding by ChIP assay and found that miR-155 knockdown eliminated the binding (Fig. 5d, right panel). Subsequent analysis revealed down-regulated KLF4 expression in both RNA (Fig. 5e, left) and protein (Fig. 5e, right) level in miR-155 knockdown cells, indicating that KLF4 is positively regulated by miR-155. In addition, a promoter assay in miRZIP155 cells with KLF4 expression revealed that KLF4 can restore the miR-155 knockdown-induced reduction in PTGES1 promoter activity (Fig. 5f and g), supporting the idea that KLF4 mediates the regulation of PTGES1 expression by miR-155. Lastly, we confirmed an elevated KLF4 expression in miR-155 high TNBC samples (Fig. 5h) consistent with our data observed from cell lines (Fig. 5e). These results collectively indicate KLF4 is a critical mediator for the regulation of PTGES1, driven by miR-155.

Even though we identified mediators for miR-155 induced PTGES1/2, we cannot exclude other possibility that can contribute to the up-regulation of PTGES1/2. As previous reports showed inverse correlation between PTGDS and PTGES [30, 31], we examined if the change of PTGDS itself affects the expression of PTGES1/2. Indeed, siRNA of PTGDS evidently up-regulates PTGES1/2 (Supplementary Figure S6b) and the overexpression of PTGDS showed the opposite effect (Supplementary Figure S6c). These data collectively suggests miR-155 up-regulates PTGES1/2 via multiple mediators.

### miR-155-mediated PGE2/PGD2 balance impact cancer cell proliferation and patients’ survival

**Fig. 6:**
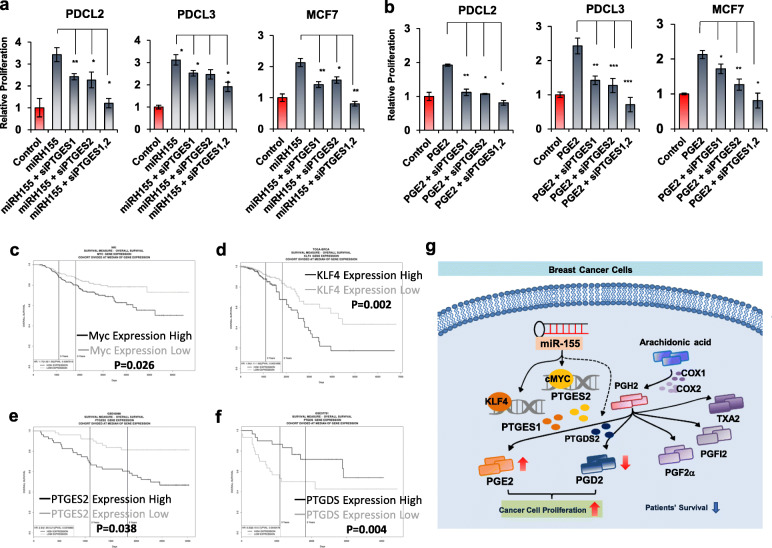
miR-155 driven prostaglandin reprogramming impact cancer cell proliferation and patients’ survival. **a**, **b** Transient knockdown of PTGES/PTGES2 restores miR-155 or PGE2-induced cellular proliferation**.** The Proliferation of miR-155-low PDC (PDCL2 and PDCL3) or MCF7 were measured after miR-155 overexpression (miRH155, **a**) or PGE2 treatment (**b**), in combination with siRNAs for PTGES/PTGES2. **c** Overexpression of PTGDS attenuates cancer cell proliferation. Two TNBC cancer cell lines were transected with PTGDS expression plasmid and cell proliferation was measured by alamarBlue assay. **d-g**. Overall survival curves of breast cancer patients determined by the expression status of cMYC(**d**), KLF4(**e**), PTGES2(**f**) and PTGDS(**g**), using the ProgeneV2 webtool. **h** Graphical abstract showing the molecular mechanism of prostaglandin reprogramming, regulated by miR-155

Accumulating evidences indicate that PGE2 exerts oncogenic function in cancer via its receptor EP receptors [12, 13] and its downstream signaling, including Ras-Raf-MEK-ERK and GSK3-beta/β-Catenin [32]. In order to delineate the functional impact of miR-155-mediated PGE2/PGD2 shift, we selected two primary breast cancer cells and MCF7 with low miR-155 expression (Supplementary Fig. S1b and S1c). We overexpressed miR-155 using lentivirus and treated them with siRNA of PTGES1 and PTGES2, to examine the contribution of those genes on miR-155 induced-oncogenicity. Cell proliferation assay results showed that miR-155 overexpression markedly increased the proliferation of the three breast cancer cells (Fig. 6a, miRH155), consistent with previous findings [21]. Importantly, we observed that the knockdown of PTGES1 or PTGES2 significantly reduced the miR-155-induced proliferation and the combination of these two siRNAs abolished the miR-155-induced proliferation in PDCL2, PDCL3 and MCF7 cells (Fig. 6a). We further confirmed the effect of PTGES1/2 knockdown in another experiment where the cell proliferation was triggered by PGE2 treatment (Fig. 6b). These data demonstrate that miR-155 exerts its oncogenicity in PTGES1/2 dependent manner. On the other hand, the overexpression expression of PTGDS significantly reduces two TNBC cell lines (Fig. 6c). To address the clinical impact of our finding, we next questioned if the expression of prostaglandin synthetase genes as well as their regulators identified in this study are associated with cancer patient’s survival. Using PROGgeneV2 webtool, we found the high expression of cMYC, KLF4, and PTGES2 are associated with poor survival whereas the high expression of PTGDS is associated with better survival in patients with breast cancer (Fig. 6d-g). Taken altogether, our results demonstrate the miR-155-mediated prostaglandin reprogramming is an important factor for the cancer cell oncogenicity as well as for the prognosis of breast cancer (Fig. 6h).

## Discussion

Several reports have shown the impact of PGE2 in breast cancer, most of which have focused on the role of Cox1 and Cox2 that catalyzes PGH2 production from arachidonic acids [33, 34]. Initial observation on the association between miR-155 and Cox expression came from the studies on immunological diseases such as asthma [22]. Later studies reported elevated levels of miR-155 and Cox2 in various cancers including colorectal cancer [35]. In this report, we could not find significant changes on COX1 or COX2 level (Figs. 1h, 2f, 3g and Supplementary Fig. 3e). TCGA analysis also showed poor correlation among miR-155, Cox-1 and Cox-2, supporting our results (Supplementary Figure S7). Instead, we revealed a role of miR-155 on prostaglandin balance via the regulation of PTGES1/2 and PTGDS, which are directly responsible for the synthesis of PGE2 and PGD2, respectively. Even though PTGES1 and PTGES2 share the same function, their localization is different: the former resides in the perinuclear zone and belongs to the MAPEG (for membrane-associated proteins involved in eicosanoid and GSH metabolism) family, whereas the latter is a Golgi membrane-associated protein, and the proteolytic removal of the N-terminal hydrophobic domain leads to the formation of a mature cytosolic enzyme [36]. The finding that miR-155 controls both enzymes suggests the broad range of functional outcome. Related to this, siRNA treatment results in the Fig. 6a-b show that the knockdown of PTGES1/2 does not fully restore the miR-155-induced proliferation, suggesting that other mechanisms of miR-155 might have induced proliferation as well as the effect of PTGDS suppressed by miR-155.

In our study, we identified KLF4 as a novel regulator for the PTGES1. A previous report showed that KLF5 regulates microsomal prostaglandin E2 synthase 1 [37]. KLF family consists of 17 members that bind to GC-rich promoter regions of the genes (consensus (G/A)(G/A)GG(C/T)G(C/T)) involved in proliferation, differentiation, and apoptosis [38]. We initially investigated KLF4 as a positively correlated gene with Fos transcription factor (Supplementary Figure S8a), which is known to be up-regulated by miR-155 [39]. Because miRNAs generally suppress its target expression, there is an inverse expression correlation between certain miRNA and its target. However, as miR-155 inhibition resulted in decreased KLF4 expression (Fig. 5e), we speculated that KLF4 is an indirect target of miR-155. Indeed, UTR analysis of KLF4 did not show any miR-155 binding sites (Supplementary Figure S8b). Interestingly, KLF4 has been shown to be upregulated by PGE2 [29]. Thus, it is possible the initial upregulation of PGE2 is driven by miR-155-Myc, triggering a positive feedback loop for the increased KLF4 expression. Another important regulator of PTGES1 is early growth response 1 (EGR1, 40), which can be induced by PGE2 and PGF2 alpha [40]. These evidences implies a network among miR-155, PTGES1 (PGE2) and EGR1 that collaborate to enhance PGE2 level.

As for PTGES2, we observed the interaction of cMYC on its promoter by miR-155 dependent manner (Fig. 4d). cMyc was previously reported to be upregulated by miR-155 via Foxo3a [19]. Thus, our results indicate both PTGES1 and 2 are indirectly up-regulated by miR-155. On the other hand, the regulation of PTGDS is not fully known. In adipocytes, one report showed that the mineralocorticoid receptor (MR) targets PTGDS [41] and another showed that PTGDS is associated with estrogen level [42]. The regulation of PTGDS by miR-155 remains unclear as well. There is no predicted miR-155 binding site in the mRNA of PTGDS (Supplementary Figure S9), so we think there are certain mediator for this regulation as well. There are known binding sites of six transcription factors on the PTGDS promoter, including HIC, ZFHX2, GLIS1, HLF, ZIC2, and EGR2 (data not shown). However, none of them are known to be regulated by miR-155 or have Ago-CLIP seq signal altered by miR-155 depletion [43]. Therefore, we speculate that other mechanisms such as epigenetic regulation or involvement of novel transcription factors are possibly responsible for this regulation.

Our study suggests that the abnormal PGE2 production in cancer cell may be reversed by inhibition of miR-155 (Fig. 3a). Most of the trials on reducing PGE2 level have been focused on the inhibition of Cox1 or Cox2, but the inhibitors showed cardiac toxicity [44], thereby preventing further development of these inhibitors in clinical trials. As the antagomiR of miR-155 (MRG-106) successfully underwent phase 1 clinical trial for lymphoid malignancy [45], our data suggest an alternative way of inhibiting PGE2 in breast cancer, by miR-155 inhibition. Additionally, a recent study showed that the Cox2/PTGES1/PGE2 pathway increases PDL1 expression in tumor-associated macrophages and MDSCs [46], suggesting that miR-155 can aid tumor immunosurveillance by stimulating PGE2 expression. Further studies are expected to clarify such regulation and to expand the use of anti-miR-155 for cancer immunotherapy.

## Conclusions

In inflammation-related cancer such as breast or lung adenocarcinoma, the up-regulated prostaglandin (especially PGE2) is regarded as pro-tumorigenic. It is attributed either cancer cell intrinsic, growth promoting activity of PGE2 via its receptor EP1–4 or the change of tumor microenvironment driven by the PGE2-responding immune cells. Thus, inhibiting PGE2 in inflammatory tumor provides an alternative therapeutic potential, in addition to the present immune checkpoint blockade inhibitors. However, the clinical trials targeting PGE2 production, which largely have focused on the inhibition of Cox1/Cox2, often showed cardiac toxicity. Our data present a valuable insight to overcome this limitation, by showing the up-regulated PGE2 production from cancer cell can be inhibited by targeting miR-155. As the antagomiR of miR-155 (MRG-106) underwent phase 1 clinical trial, its benefit should be considered in the context of prostaglandin metabolism of cancer.

## Supplementary Information

**Additional file 1.**

## Data Availability

All data generated or analyzed during this study are included in this published article and its supplementary information files.

## Abbreviations

PGE2: Prostaglandin E2
PGD2: Prostaglandin D2
TNBC: Triple negative breast cancer
PTGES: Prostaglandin E2 synthase
PTGDS: Prostaglandin D2 synthase
15-PGDH: 15-Hydroxyprostaglandin dehydrogenase
COX-2: Cyclooxigenase-2
PDCs: Patient derived cells
