# Supplementary material for: Targeted eicosanoids profiling reveals a prostaglandin reprogramming in breast Cancer by microRNA-155
Source: J Exp Clin Cancer Res. 2021 Jan 25;40:43. doi: 10.1186/s13046-021-01839-4 (PMC7831268; doi:10.1186/s13046-021-01839-4)
Supplement: Supplementary file 1 — Additional file 1. [file 13046_2021_1839_MOESM1_ESM.pptx]

## Slide 1
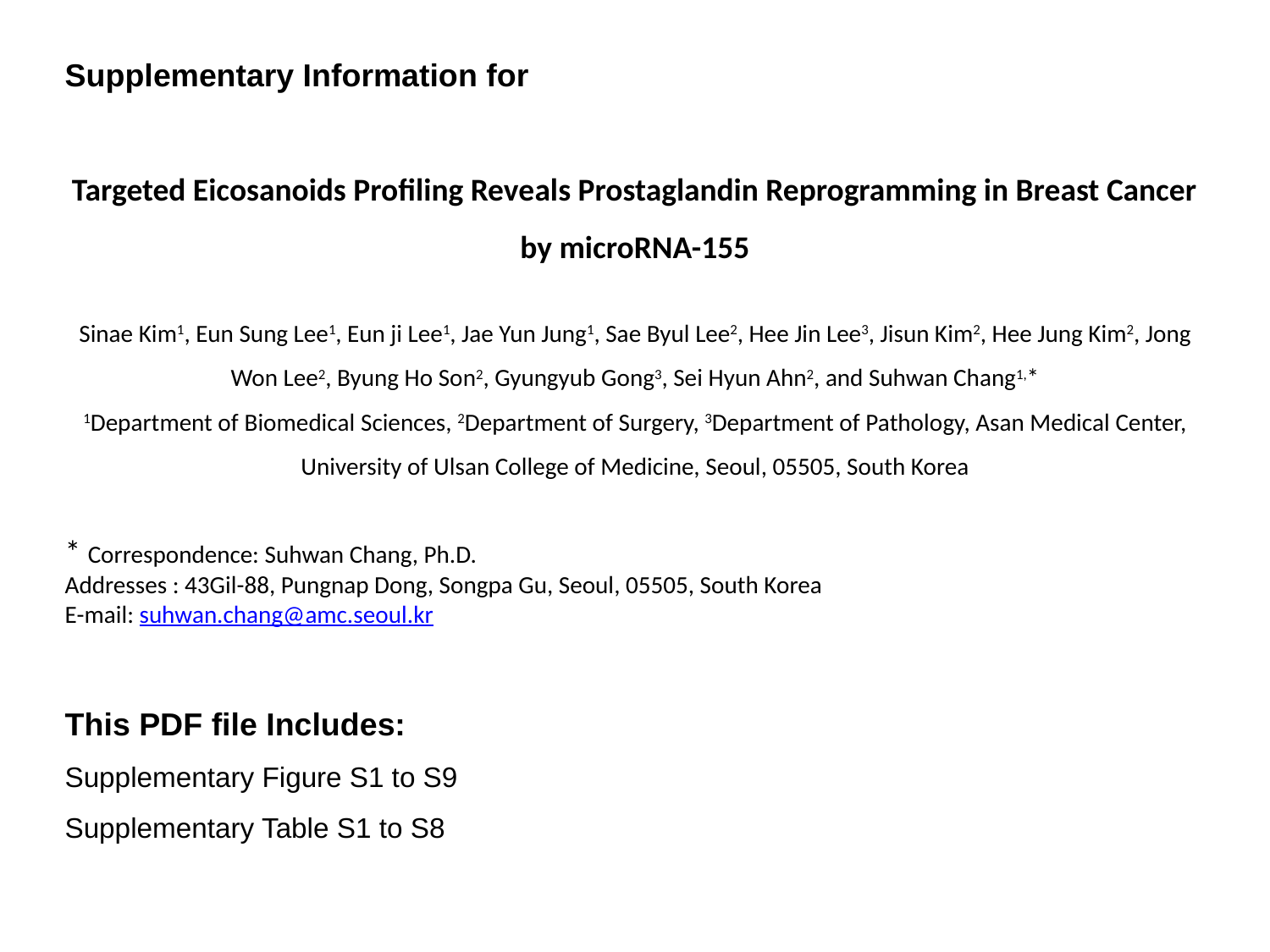

Supplementary Information for
Targeted Eicosanoids Profiling Reveals Prostaglandin Reprogramming in Breast Cancer by microRNA-155
Sinae Kim1, Eun Sung Lee1, Eun ji Lee1, Jae Yun Jung1, Sae Byul Lee2, Hee Jin Lee3, Jisun Kim2, Hee Jung Kim2, Jong Won Lee2, Byung Ho Son2, Gyungyub Gong3, Sei Hyun Ahn2, and Suhwan Chang1,*
1Department of Biomedical Sciences, 2Department of Surgery, 3Department of Pathology, Asan Medical Center, University of Ulsan College of Medicine, Seoul, 05505, South Korea
* Correspondence: Suhwan Chang, Ph.D.
Addresses : 43Gil-88, Pungnap Dong, Songpa Gu, Seoul, 05505, South Korea
E-mail: suhwan.chang@amc.seoul.kr
This PDF file Includes:
Supplementary Figure S1 to S9
Supplementary Table S1 to S8

## Slide 2
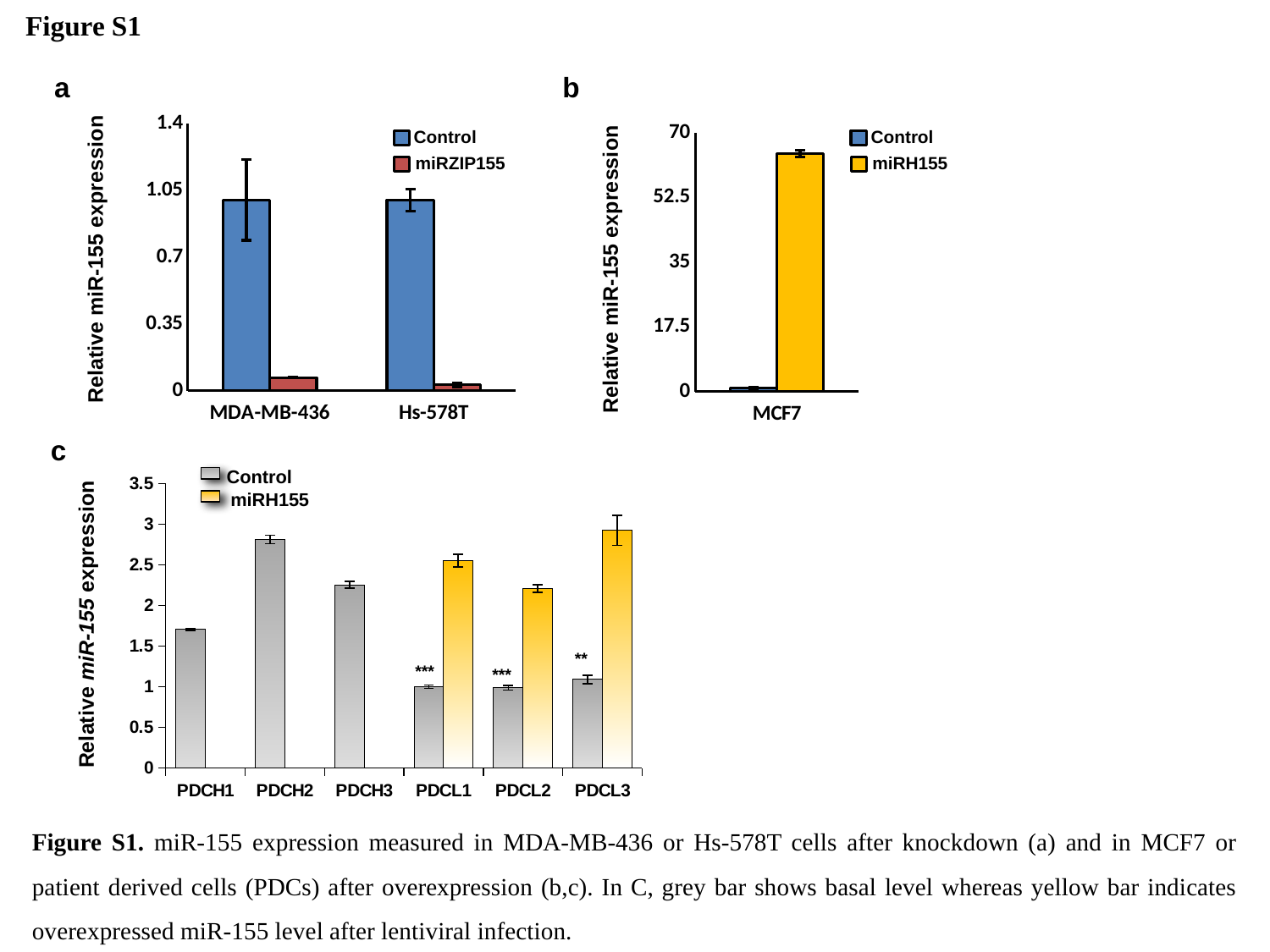

Figure S1
a
b
### Chart
| Category | Control | miRZIP155 |
|---|---|---|
| MDA-MB-436 | 1.0 | 0.068537 |
| Hs-578T | 1.0 | 0.030666 |
### Chart
| Category | Control | miRZIP155 |
|---|---|---|
| MCF7 | 1.0 | 64.426384 |Control
Control
miRZIP155
miRH155
Relative miR-155 expression
Relative miR-155 expression
c
 Control
 miRH155
### Chart
| Category | | |
|---|---|---|
| PDCH1 | 1.7050508216544464 | None |
| PDCH2 | 2.815281981526469 | None |
| PDCH3 | 2.255237592321676 | None |
| PDCL1 | 1.0 | 2.555416733138502 |
| PDCL2 | 0.9886650782767209 | 2.208895483970928 |
| PDCL3 | 1.0899330628574397 | 2.924981884714295 |Relative miR-155 expression
**
***
***
Figure S1. miR-155 expression measured in MDA-MB-436 or Hs-578T cells after knockdown (a) and in MCF7 or patient derived cells (PDCs) after overexpression (b,c). In C, grey bar shows basal level whereas yellow bar indicates overexpressed miR-155 level after lentiviral infection.

## Slide 3
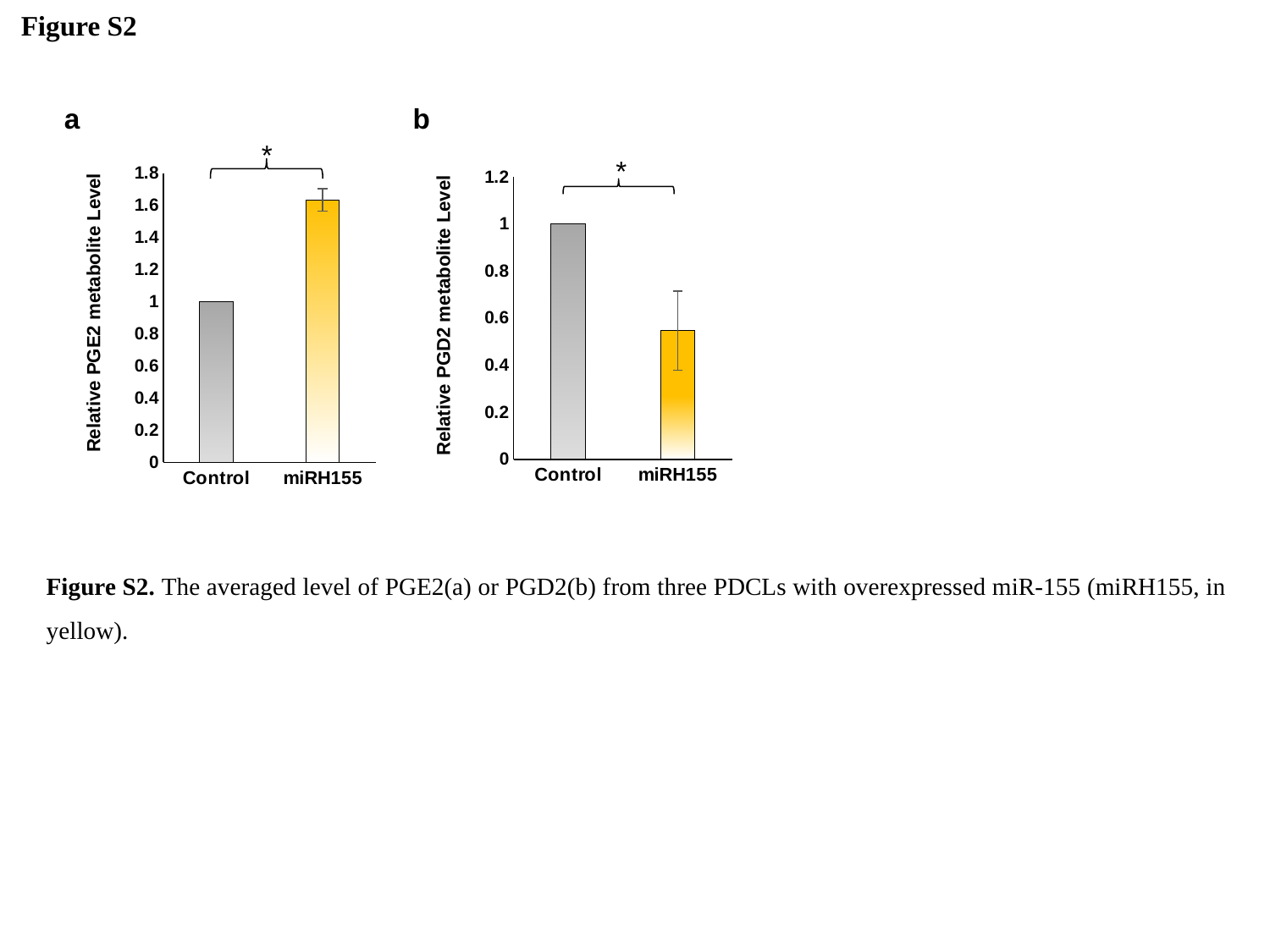

Figure S2
a
b
*
*
### Chart
| Category | |
|---|---|
| Control | 1.0 |
| miRH155 | 0.5464309292604994 |
### Chart
| Category | |
|---|---|
| Control | 1.0 |
| miRH155 | 1.634045680687472 |Relative PGD2 metabolite Level
Relative PGE2 metabolite Level
Figure S2. The averaged level of PGE2(a) or PGD2(b) from three PDCLs with overexpressed miR-155 (miRH155, in yellow).

## Slide 4
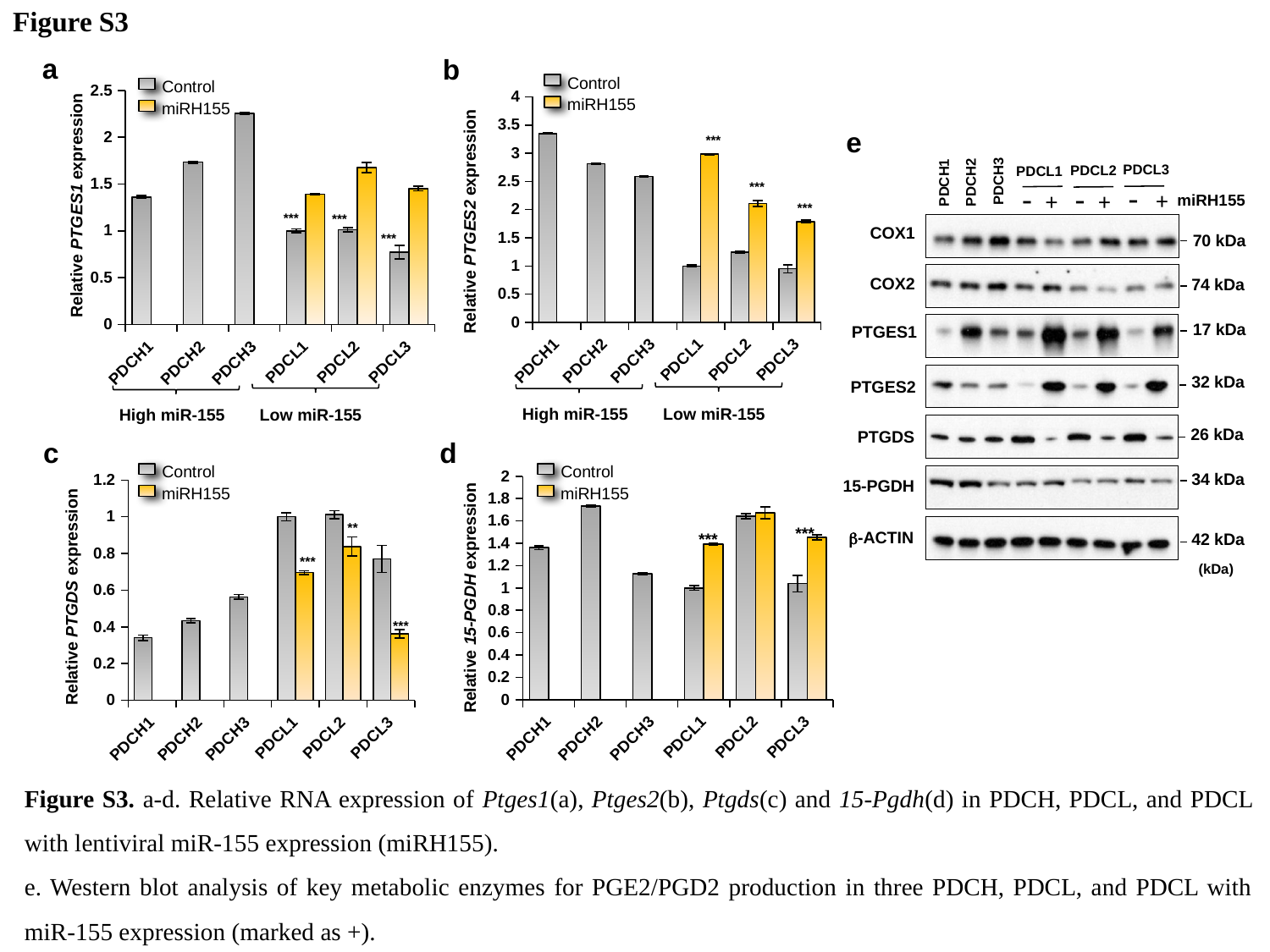

Figure S3
a
b
 Control
 miRH155
 Control
 miRH155
### Chart
| Category | | |
|---|---|---|
| PDCH1 | 1.3606638957935286 | None |
| PDCH2 | 1.7330093417734582 | None |
| PDCH3 | 2.255237592321675 | None |
| PDCL1 | 1.0 | 1.391704263938145 |
| PDCL2 | 1.011505752571905 | 1.675926328553106 |
| PDCL3 | 0.770699059785919 | 1.4519253266580643 |
### Chart
| Category | | |
|---|---|---|
| PDCH1 | 3.3503475074926654 | None |
| PDCH2 | 2.815281981526471 | None |
| PDCH3 | 2.590587712427383 | None |
| PDCL1 | 1.0000000000000002 | 2.9831833955750184 |
| PDCL2 | 1.2453096563451462 | 2.1102729626818677 |
| PDCL3 | 0.9488418418256143 | 1.787529754509069 |e
***
PDCL3
-
+
PDCL2
-
+
PDCL1
-
+
***
PDCH3
PDCH1
PDCH2
miRH155
Relative PTGES1 expression
***
***
Relative PTGES2 expression
***
COX1
***
70 kDa
COX2
74 kDa
17 kDa
PTGES1
32 kDa
PTGES2
High miR-155
Low miR-155
High miR-155
Low miR-155
26 kDa
PTGDS
c
d
 Control
 miRH155
 Control
 miRH155
### Chart
| Category | | |
|---|---|---|
| PDCH1 | 1.3606638957935286 | None |
| PDCH2 | 1.7330093417734582 | None |
| PDCH3 | 1.1276187961608375 | None |
| PDCL1 | 1.0 | 1.391704263938145 |
| PDCL2 | 1.6431959429092948 | 1.6740297393723698 |
| PDCL3 | 1.0399430499613258 | 1.4519253266580643 |
### Chart
| Category | | |
|---|---|---|
| PDCH1 | 0.3401659739483824 | None |
| PDCH2 | 0.4332523354433646 | None |
| PDCH3 | 0.5638093980804189 | None |
| PDCL1 | 1.0 | 0.6958521319690725 |
| PDCL2 | 1.0115057525719047 | 0.8379631642765532 |
| PDCL3 | 0.7706990597859193 | 0.3629813316645161 |34 kDa
15-PGDH
**
***
b-ACTIN
***
42 kDa
***
(kDa)
Relative PTGDS expression
Relative 15-PGDH expression
***
Figure S3. a-d. Relative RNA expression of Ptges1(a), Ptges2(b), Ptgds(c) and 15-Pgdh(d) in PDCH, PDCL, and PDCL with lentiviral miR-155 expression (miRH155).
e. Western blot analysis of key metabolic enzymes for PGE2/PGD2 production in three PDCH, PDCL, and PDCL with miR-155 expression (marked as +).

## Slide 5
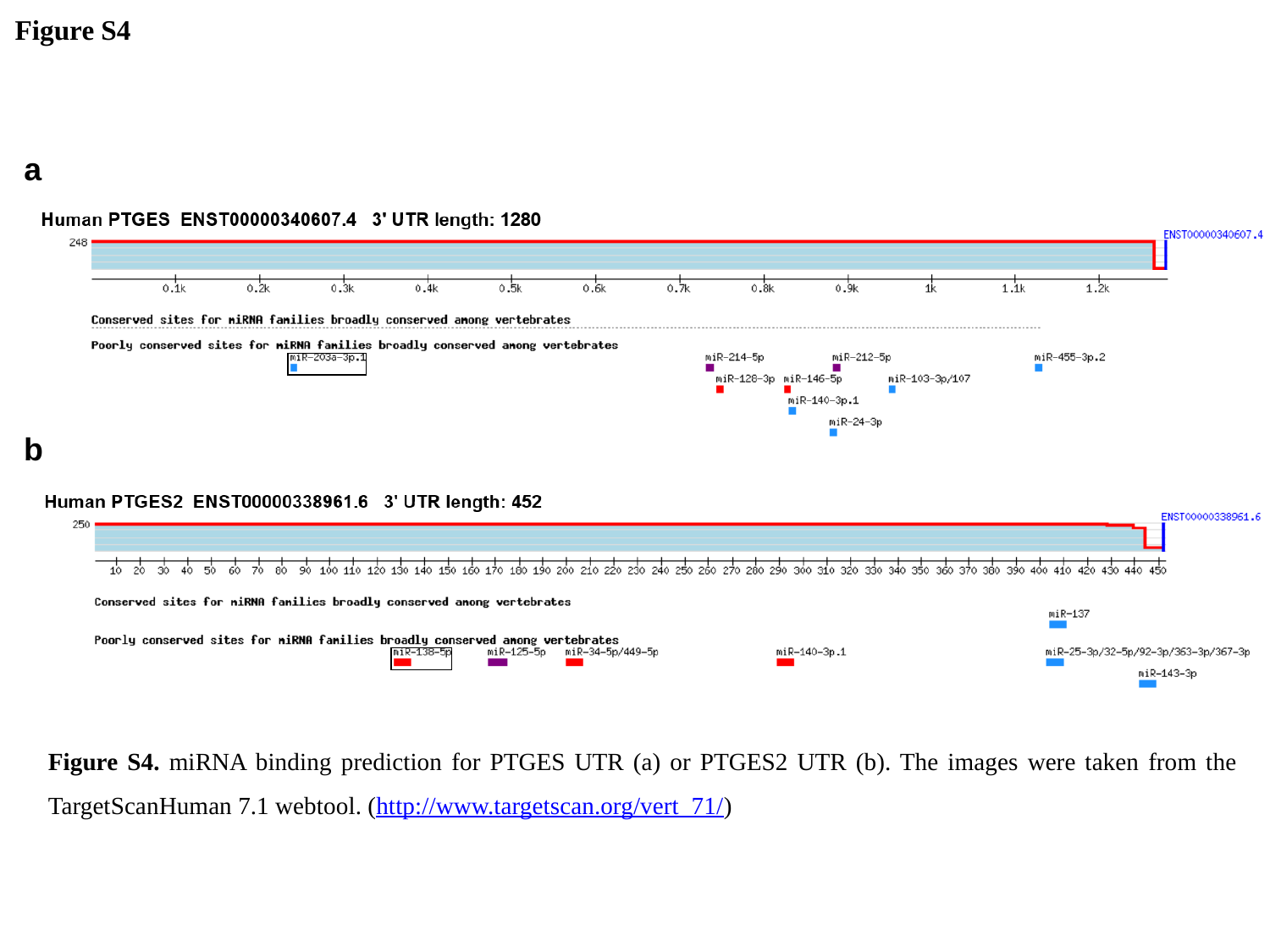

Figure S4
a
b
Figure S4. miRNA binding prediction for PTGES UTR (a) or PTGES2 UTR (b). The images were taken from the TargetScanHuman 7.1 webtool. (http://www.targetscan.org/vert_71/)

## Slide 6
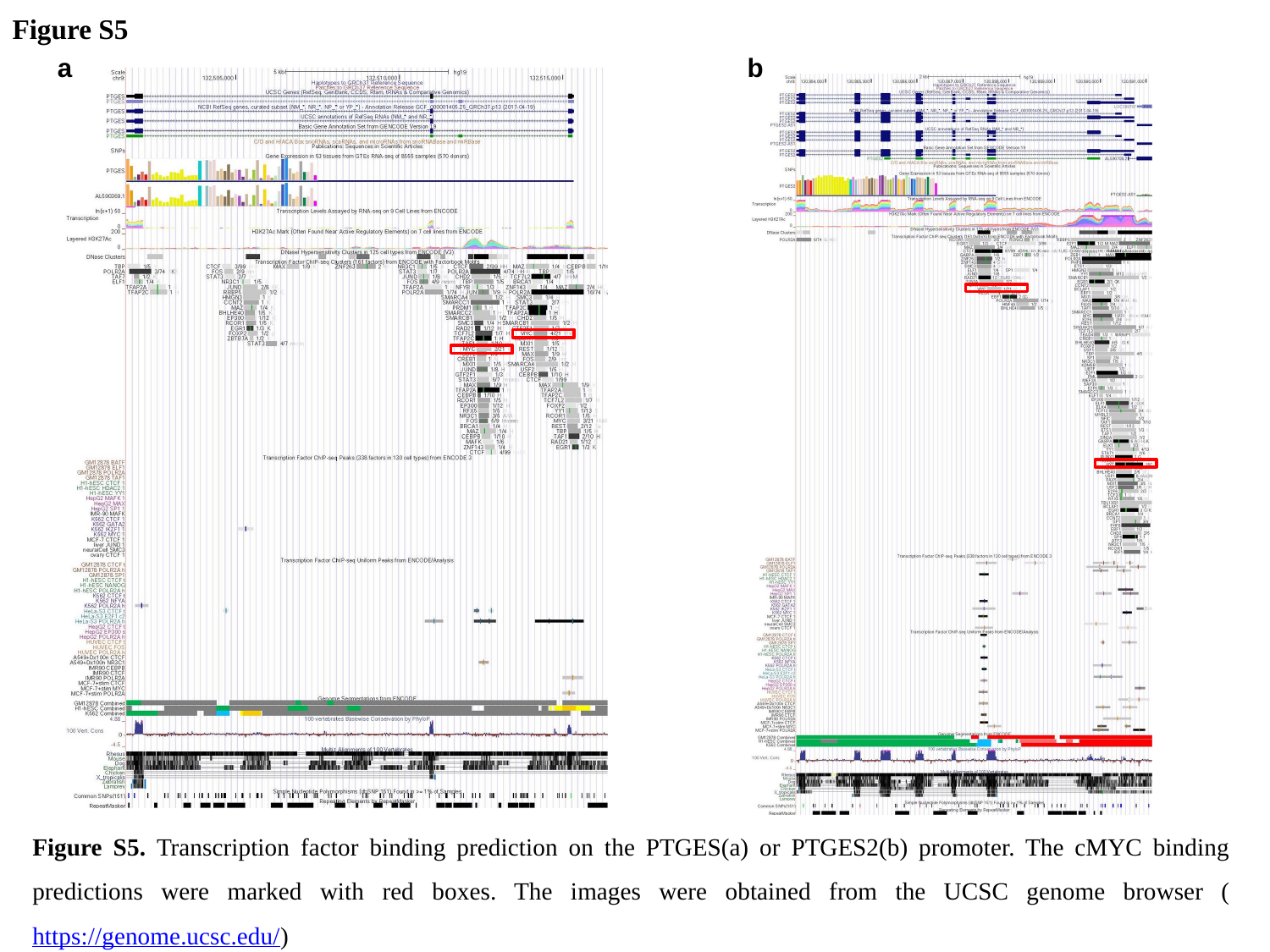

Figure S5
a
b
Figure S5. Transcription factor binding prediction on the PTGES(a) or PTGES2(b) promoter. The cMYC binding predictions were marked with red boxes. The images were obtained from the UCSC genome browser (https://genome.ucsc.edu/)

## Slide 7
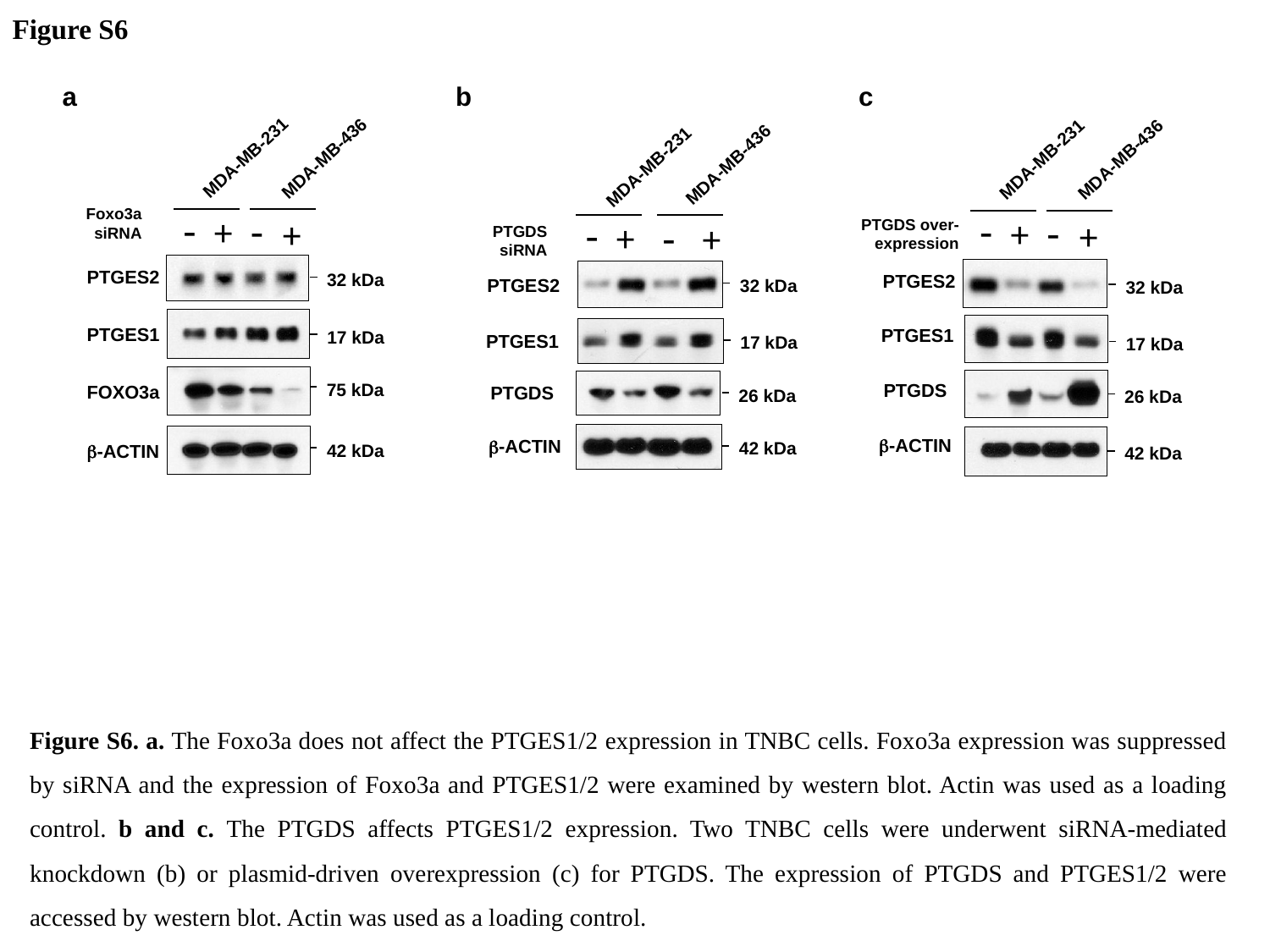

Figure S6
a
b
c
MDA-MB-231
MDA-MB-436
MDA-MB-231
MDA-MB-436
MDA-MB-436
MDA-MB-231
Foxo3a siRNA
-
-
-
-
+
-
+
-
+
+
PTGDS over-expression
+
+
PTGDS siRNA
PTGES2
32 kDa
PTGES2
PTGES2
32 kDa
32 kDa
PTGES1
PTGES1
17 kDa
PTGES1
17 kDa
17 kDa
75 kDa
PTGDS
FOXO3a
PTGDS
26 kDa
26 kDa
b-ACTIN
b-ACTIN
42 kDa
42 kDa
b-ACTIN
42 kDa
Figure S6. a. The Foxo3a does not affect the PTGES1/2 expression in TNBC cells. Foxo3a expression was suppressed by siRNA and the expression of Foxo3a and PTGES1/2 were examined by western blot. Actin was used as a loading control. b and c. The PTGDS affects PTGES1/2 expression. Two TNBC cells were underwent siRNA-mediated knockdown (b) or plasmid-driven overexpression (c) for PTGDS. The expression of PTGDS and PTGES1/2 were accessed by western blot. Actin was used as a loading control.

## Slide 8
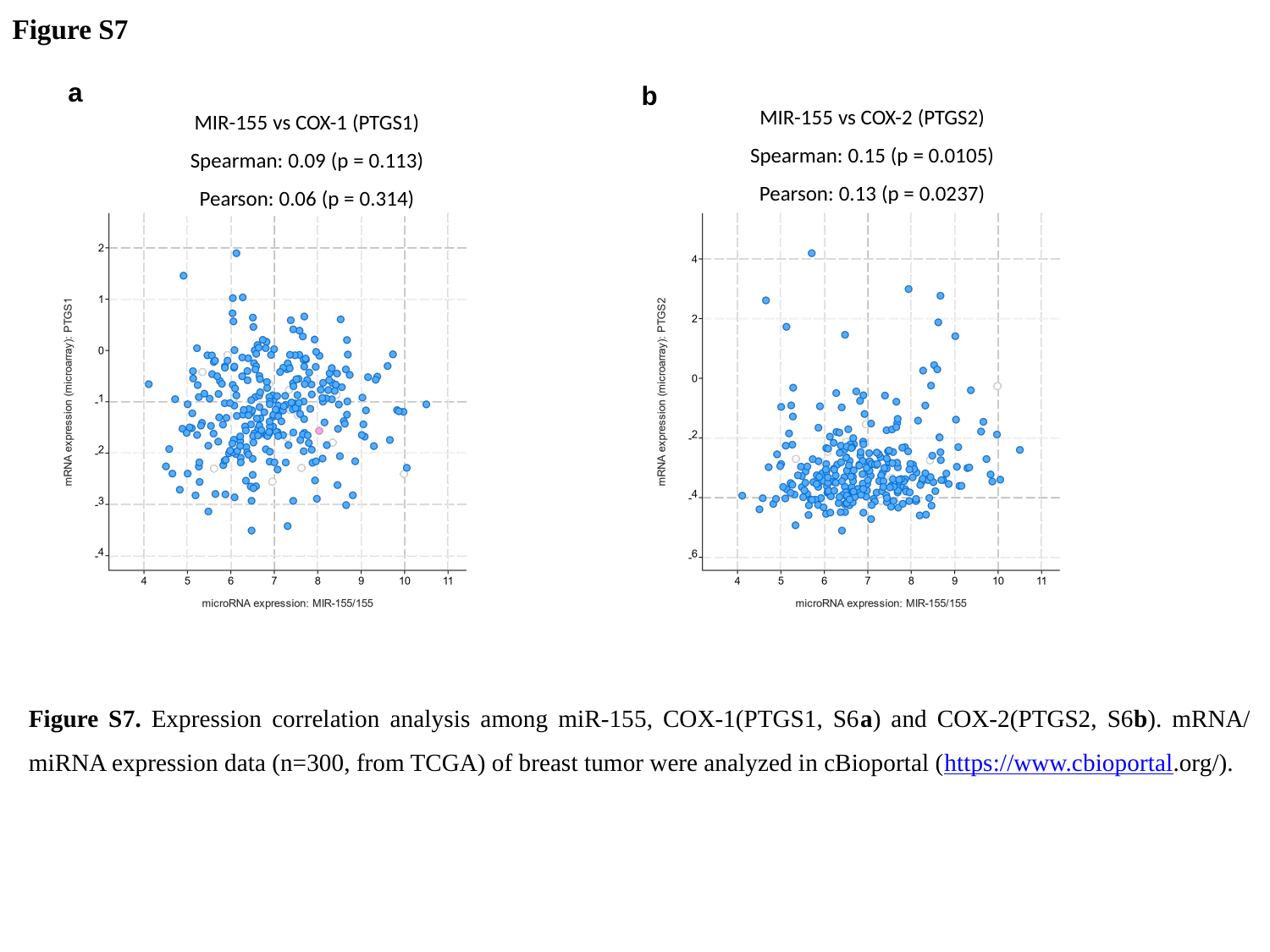

Figure S7
a
b
MIR-155 vs COX-2 (PTGS2)
Spearman: 0.15 (p = 0.0105)
Pearson: 0.13 (p = 0.0237)
MIR-155 vs COX-1 (PTGS1)
Spearman: 0.09 (p = 0.113)
Pearson: 0.06 (p = 0.314)
Figure S7. Expression correlation analysis among miR-155, COX-1(PTGS1, S6a) and COX-2(PTGS2, S6b). mRNA/ miRNA expression data (n=300, from TCGA) of breast tumor were analyzed in cBioportal (https://www.cbioportal.org/).

## Slide 9
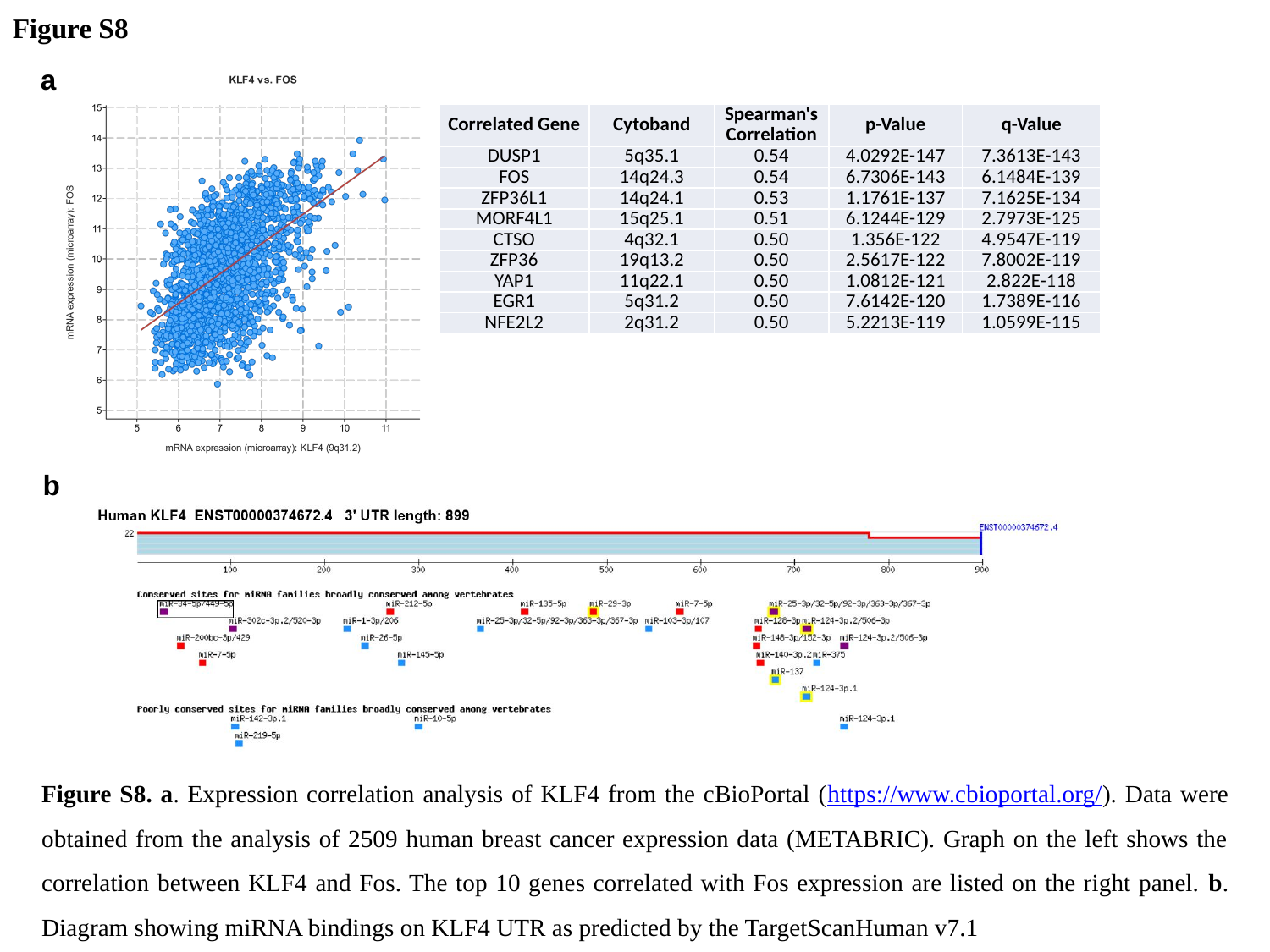

Figure S8
a
| Correlated Gene | Cytoband | Spearman's Correlation | p-Value | q-Value |
| --- | --- | --- | --- | --- |
| DUSP1 | 5q35.1 | 0.54 | 4.0292E-147 | 7.3613E-143 |
| FOS | 14q24.3 | 0.54 | 6.7306E-143 | 6.1484E-139 |
| ZFP36L1 | 14q24.1 | 0.53 | 1.1761E-137 | 7.1625E-134 |
| MORF4L1 | 15q25.1 | 0.51 | 6.1244E-129 | 2.7973E-125 |
| CTSO | 4q32.1 | 0.50 | 1.356E-122 | 4.9547E-119 |
| ZFP36 | 19q13.2 | 0.50 | 2.5617E-122 | 7.8002E-119 |
| YAP1 | 11q22.1 | 0.50 | 1.0812E-121 | 2.822E-118 |
| EGR1 | 5q31.2 | 0.50 | 7.6142E-120 | 1.7389E-116 |
| NFE2L2 | 2q31.2 | 0.50 | 5.2213E-119 | 1.0599E-115 |
b
Figure S8. a. Expression correlation analysis of KLF4 from the cBioPortal (https://www.cbioportal.org/). Data were obtained from the analysis of 2509 human breast cancer expression data (METABRIC). Graph on the left shows the correlation between KLF4 and Fos. The top 10 genes correlated with Fos expression are listed on the right panel. b. Diagram showing miRNA bindings on KLF4 UTR as predicted by the TargetScanHuman v7.1

## Slide 10
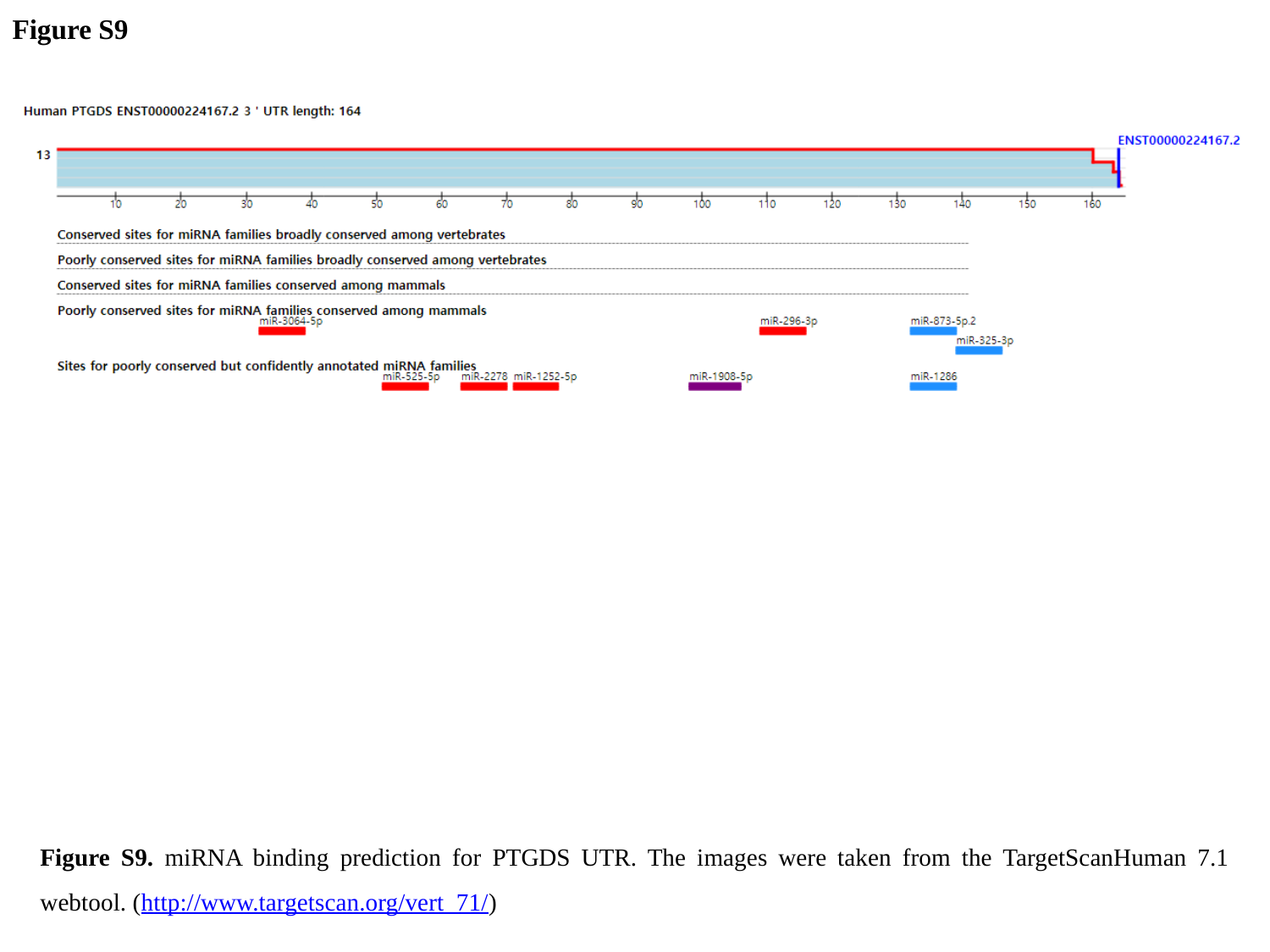

Figure S9
Figure S9. miRNA binding prediction for PTGDS UTR. The images were taken from the TargetScanHuman 7.1 webtool. (http://www.targetscan.org/vert_71/)

## Slide 11
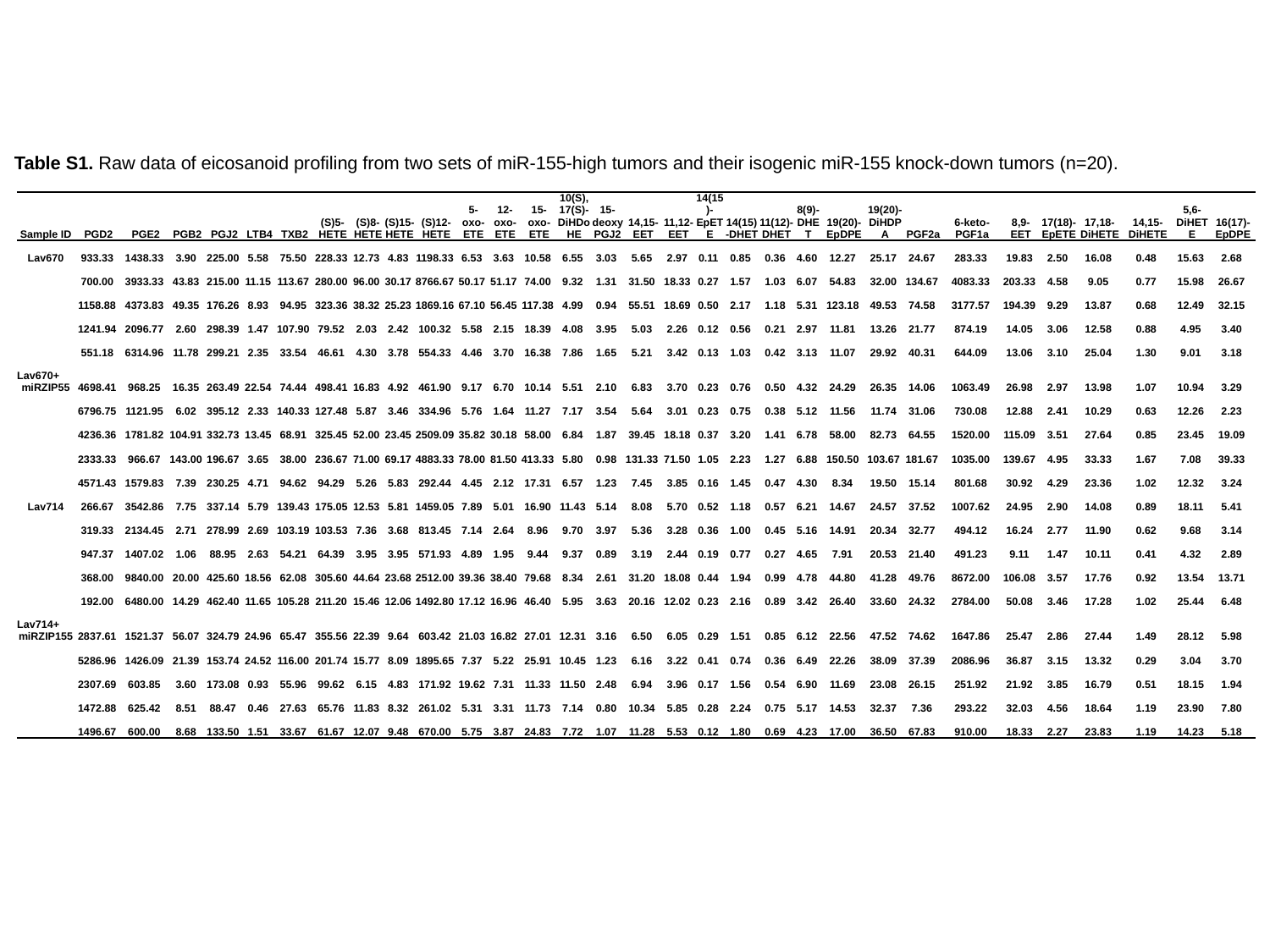

Table S1. Raw data of eicosanoid profiling from two sets of miR-155-high tumors and their isogenic miR-155 knock-down tumors (n=20).
| Sample ID | PGD2 | PGE2 | PGB2 | PGJ2 | LTB4 | TXB2 | (S)5-HETE | (S)8-HETE | (S)15-HETE | (S)12-HETE | 5-oxo-ETE | 12-oxo-ETE | 15-oxo-ETE | 10(S),17(S)-DiHDoHE | 15-deoxy PGJ2 | 14,15-EET | 11,12-EET | 14(15)-EpETE | 14(15)-DHET | 11(12)-DHET | 8(9)-DHET | 19(20)-EpDPE | 19(20)-DiHDPA | PGF2a | 6-keto-PGF1a | 8,9-EET | 17(18)-EpETE | 17,18-DiHETE | 14,15-DiHETE | 5,6-DiHETE | 16(17)-EpDPE |
| --- | --- | --- | --- | --- | --- | --- | --- | --- | --- | --- | --- | --- | --- | --- | --- | --- | --- | --- | --- | --- | --- | --- | --- | --- | --- | --- | --- | --- | --- | --- | --- |
| Lav670 | 933.33 | 1438.33 | 3.90 | 225.00 | 5.58 | 75.50 | 228.33 | 12.73 | 4.83 | 1198.33 | 6.53 | 3.63 | 10.58 | 6.55 | 3.03 | 5.65 | 2.97 | 0.11 | 0.85 | 0.36 | 4.60 | 12.27 | 25.17 | 24.67 | 283.33 | 19.83 | 2.50 | 16.08 | 0.48 | 15.63 | 2.68 |
| | 700.00 | 3933.33 | 43.83 | 215.00 | 11.15 | 113.67 | 280.00 | 96.00 | 30.17 | 8766.67 | 50.17 | 51.17 | 74.00 | 9.32 | 1.31 | 31.50 | 18.33 | 0.27 | 1.57 | 1.03 | 6.07 | 54.83 | 32.00 | 134.67 | 4083.33 | 203.33 | 4.58 | 9.05 | 0.77 | 15.98 | 26.67 |
| | 1158.88 | 4373.83 | 49.35 | 176.26 | 8.93 | 94.95 | 323.36 | 38.32 | 25.23 | 1869.16 | 67.10 | 56.45 | 117.38 | 4.99 | 0.94 | 55.51 | 18.69 | 0.50 | 2.17 | 1.18 | 5.31 | 123.18 | 49.53 | 74.58 | 3177.57 | 194.39 | 9.29 | 13.87 | 0.68 | 12.49 | 32.15 |
| | 1241.94 | 2096.77 | 2.60 | 298.39 | 1.47 | 107.90 | 79.52 | 2.03 | 2.42 | 100.32 | 5.58 | 2.15 | 18.39 | 4.08 | 3.95 | 5.03 | 2.26 | 0.12 | 0.56 | 0.21 | 2.97 | 11.81 | 13.26 | 21.77 | 874.19 | 14.05 | 3.06 | 12.58 | 0.88 | 4.95 | 3.40 |
| | 551.18 | 6314.96 | 11.78 | 299.21 | 2.35 | 33.54 | 46.61 | 4.30 | 3.78 | 554.33 | 4.46 | 3.70 | 16.38 | 7.86 | 1.65 | 5.21 | 3.42 | 0.13 | 1.03 | 0.42 | 3.13 | 11.07 | 29.92 | 40.31 | 644.09 | 13.06 | 3.10 | 25.04 | 1.30 | 9.01 | 3.18 |
| Lav670+ miRZIP55 | 4698.41 | 968.25 | 16.35 | 263.49 | 22.54 | 74.44 | 498.41 | 16.83 | 4.92 | 461.90 | 9.17 | 6.70 | 10.14 | 5.51 | 2.10 | 6.83 | 3.70 | 0.23 | 0.76 | 0.50 | 4.32 | 24.29 | 26.35 | 14.06 | 1063.49 | 26.98 | 2.97 | 13.98 | 1.07 | 10.94 | 3.29 |
| | 6796.75 | 1121.95 | 6.02 | 395.12 | 2.33 | 140.33 | 127.48 | 5.87 | 3.46 | 334.96 | 5.76 | 1.64 | 11.27 | 7.17 | 3.54 | 5.64 | 3.01 | 0.23 | 0.75 | 0.38 | 5.12 | 11.56 | 11.74 | 31.06 | 730.08 | 12.88 | 2.41 | 10.29 | 0.63 | 12.26 | 2.23 |
| | 4236.36 | 1781.82 | 104.91 | 332.73 | 13.45 | 68.91 | 325.45 | 52.00 | 23.45 | 2509.09 | 35.82 | 30.18 | 58.00 | 6.84 | 1.87 | 39.45 | 18.18 | 0.37 | 3.20 | 1.41 | 6.78 | 58.00 | 82.73 | 64.55 | 1520.00 | 115.09 | 3.51 | 27.64 | 0.85 | 23.45 | 19.09 |
| | 2333.33 | 966.67 | 143.00 | 196.67 | 3.65 | 38.00 | 236.67 | 71.00 | 69.17 | 4883.33 | 78.00 | 81.50 | 413.33 | 5.80 | 0.98 | 131.33 | 71.50 | 1.05 | 2.23 | 1.27 | 6.88 | 150.50 | 103.67 | 181.67 | 1035.00 | 139.67 | 4.95 | 33.33 | 1.67 | 7.08 | 39.33 |
| | 4571.43 | 1579.83 | 7.39 | 230.25 | 4.71 | 94.62 | 94.29 | 5.26 | 5.83 | 292.44 | 4.45 | 2.12 | 17.31 | 6.57 | 1.23 | 7.45 | 3.85 | 0.16 | 1.45 | 0.47 | 4.30 | 8.34 | 19.50 | 15.14 | 801.68 | 30.92 | 4.29 | 23.36 | 1.02 | 12.32 | 3.24 |
| Lav714 | 266.67 | 3542.86 | 7.75 | 337.14 | 5.79 | 139.43 | 175.05 | 12.53 | 5.81 | 1459.05 | 7.89 | 5.01 | 16.90 | 11.43 | 5.14 | 8.08 | 5.70 | 0.52 | 1.18 | 0.57 | 6.21 | 14.67 | 24.57 | 37.52 | 1007.62 | 24.95 | 2.90 | 14.08 | 0.89 | 18.11 | 5.41 |
| | 319.33 | 2134.45 | 2.71 | 278.99 | 2.69 | 103.19 | 103.53 | 7.36 | 3.68 | 813.45 | 7.14 | 2.64 | 8.96 | 9.70 | 3.97 | 5.36 | 3.28 | 0.36 | 1.00 | 0.45 | 5.16 | 14.91 | 20.34 | 32.77 | 494.12 | 16.24 | 2.77 | 11.90 | 0.62 | 9.68 | 3.14 |
| | 947.37 | 1407.02 | 1.06 | 88.95 | 2.63 | 54.21 | 64.39 | 3.95 | 3.95 | 571.93 | 4.89 | 1.95 | 9.44 | 9.37 | 0.89 | 3.19 | 2.44 | 0.19 | 0.77 | 0.27 | 4.65 | 7.91 | 20.53 | 21.40 | 491.23 | 9.11 | 1.47 | 10.11 | 0.41 | 4.32 | 2.89 |
| | 368.00 | 9840.00 | 20.00 | 425.60 | 18.56 | 62.08 | 305.60 | 44.64 | 23.68 | 2512.00 | 39.36 | 38.40 | 79.68 | 8.34 | 2.61 | 31.20 | 18.08 | 0.44 | 1.94 | 0.99 | 4.78 | 44.80 | 41.28 | 49.76 | 8672.00 | 106.08 | 3.57 | 17.76 | 0.92 | 13.54 | 13.71 |
| | 192.00 | 6480.00 | 14.29 | 462.40 | 11.65 | 105.28 | 211.20 | 15.46 | 12.06 | 1492.80 | 17.12 | 16.96 | 46.40 | 5.95 | 3.63 | 20.16 | 12.02 | 0.23 | 2.16 | 0.89 | 3.42 | 26.40 | 33.60 | 24.32 | 2784.00 | 50.08 | 3.46 | 17.28 | 1.02 | 25.44 | 6.48 |
| Lav714+ miRZIP155 | 2837.61 | 1521.37 | 56.07 | 324.79 | 24.96 | 65.47 | 355.56 | 22.39 | 9.64 | 603.42 | 21.03 | 16.82 | 27.01 | 12.31 | 3.16 | 6.50 | 6.05 | 0.29 | 1.51 | 0.85 | 6.12 | 22.56 | 47.52 | 74.62 | 1647.86 | 25.47 | 2.86 | 27.44 | 1.49 | 28.12 | 5.98 |
| | 5286.96 | 1426.09 | 21.39 | 153.74 | 24.52 | 116.00 | 201.74 | 15.77 | 8.09 | 1895.65 | 7.37 | 5.22 | 25.91 | 10.45 | 1.23 | 6.16 | 3.22 | 0.41 | 0.74 | 0.36 | 6.49 | 22.26 | 38.09 | 37.39 | 2086.96 | 36.87 | 3.15 | 13.32 | 0.29 | 3.04 | 3.70 |
| | 2307.69 | 603.85 | 3.60 | 173.08 | 0.93 | 55.96 | 99.62 | 6.15 | 4.83 | 171.92 | 19.62 | 7.31 | 11.33 | 11.50 | 2.48 | 6.94 | 3.96 | 0.17 | 1.56 | 0.54 | 6.90 | 11.69 | 23.08 | 26.15 | 251.92 | 21.92 | 3.85 | 16.79 | 0.51 | 18.15 | 1.94 |
| | 1472.88 | 625.42 | 8.51 | 88.47 | 0.46 | 27.63 | 65.76 | 11.83 | 8.32 | 261.02 | 5.31 | 3.31 | 11.73 | 7.14 | 0.80 | 10.34 | 5.85 | 0.28 | 2.24 | 0.75 | 5.17 | 14.53 | 32.37 | 7.36 | 293.22 | 32.03 | 4.56 | 18.64 | 1.19 | 23.90 | 7.80 |
| | 1496.67 | 600.00 | 8.68 | 133.50 | 1.51 | 33.67 | 61.67 | 12.07 | 9.48 | 670.00 | 5.75 | 3.87 | 24.83 | 7.72 | 1.07 | 11.28 | 5.53 | 0.12 | 1.80 | 0.69 | 4.23 | 17.00 | 36.50 | 67.83 | 910.00 | 18.33 | 2.27 | 23.83 | 1.19 | 14.23 | 5.18 |

## Slide 12
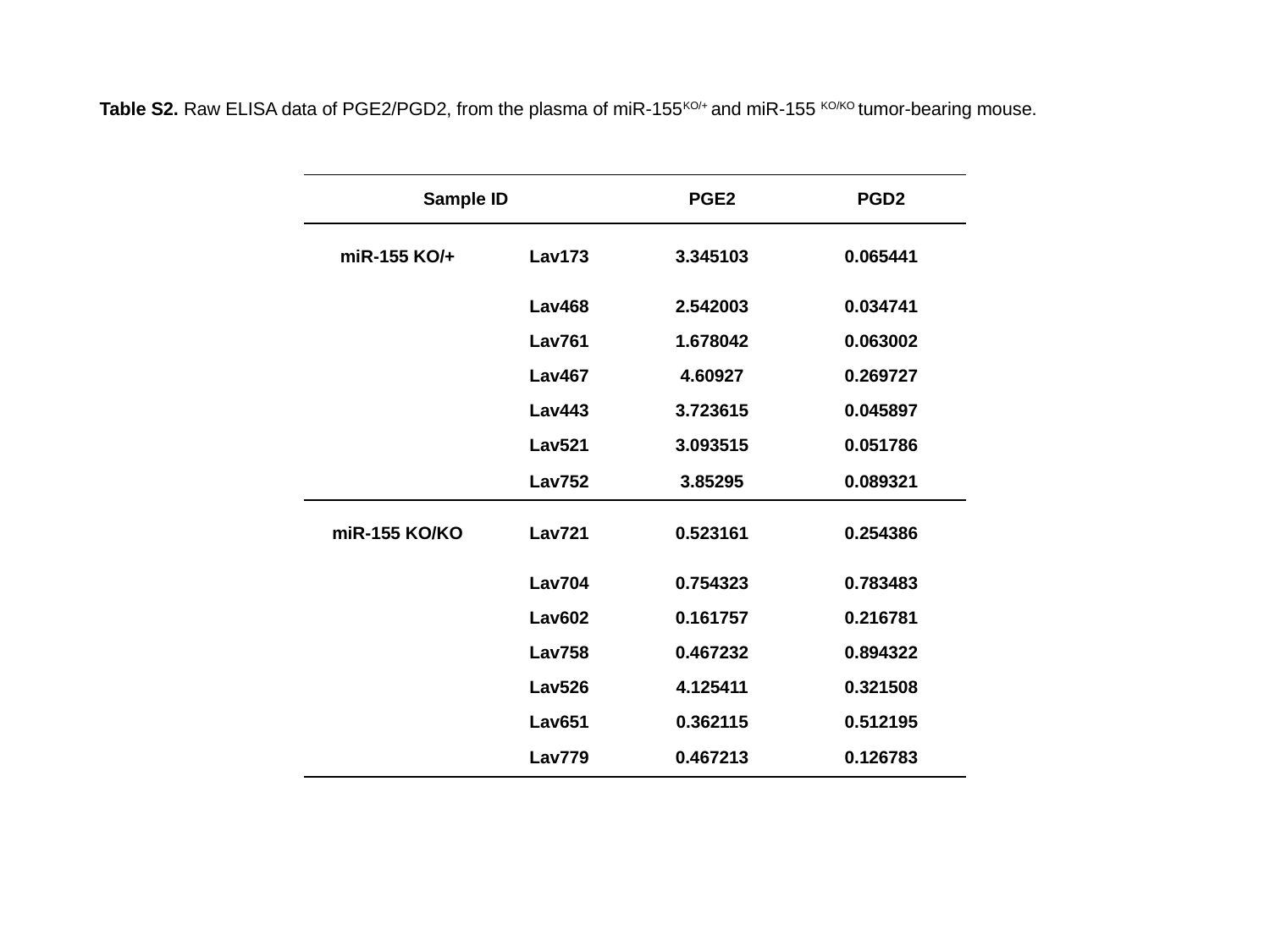

Table S2. Raw ELISA data of PGE2/PGD2, from the plasma of miR-155KO/+ and miR-155 KO/KO tumor-bearing mouse.
| Sample ID | | PGE2 | PGD2 |
| --- | --- | --- | --- |
| miR-155 KO/+ | Lav173 | 3.345103 | 0.065441 |
| | Lav468 | 2.542003 | 0.034741 |
| | Lav761 | 1.678042 | 0.063002 |
| | Lav467 | 4.60927 | 0.269727 |
| | Lav443 | 3.723615 | 0.045897 |
| | Lav521 | 3.093515 | 0.051786 |
| | Lav752 | 3.85295 | 0.089321 |
| miR-155 KO/KO | Lav721 | 0.523161 | 0.254386 |
| | Lav704 | 0.754323 | 0.783483 |
| | Lav602 | 0.161757 | 0.216781 |
| | Lav758 | 0.467232 | 0.894322 |
| | Lav526 | 4.125411 | 0.321508 |
| | Lav651 | 0.362115 | 0.512195 |
| | Lav779 | 0.467213 | 0.126783 |

## Slide 13
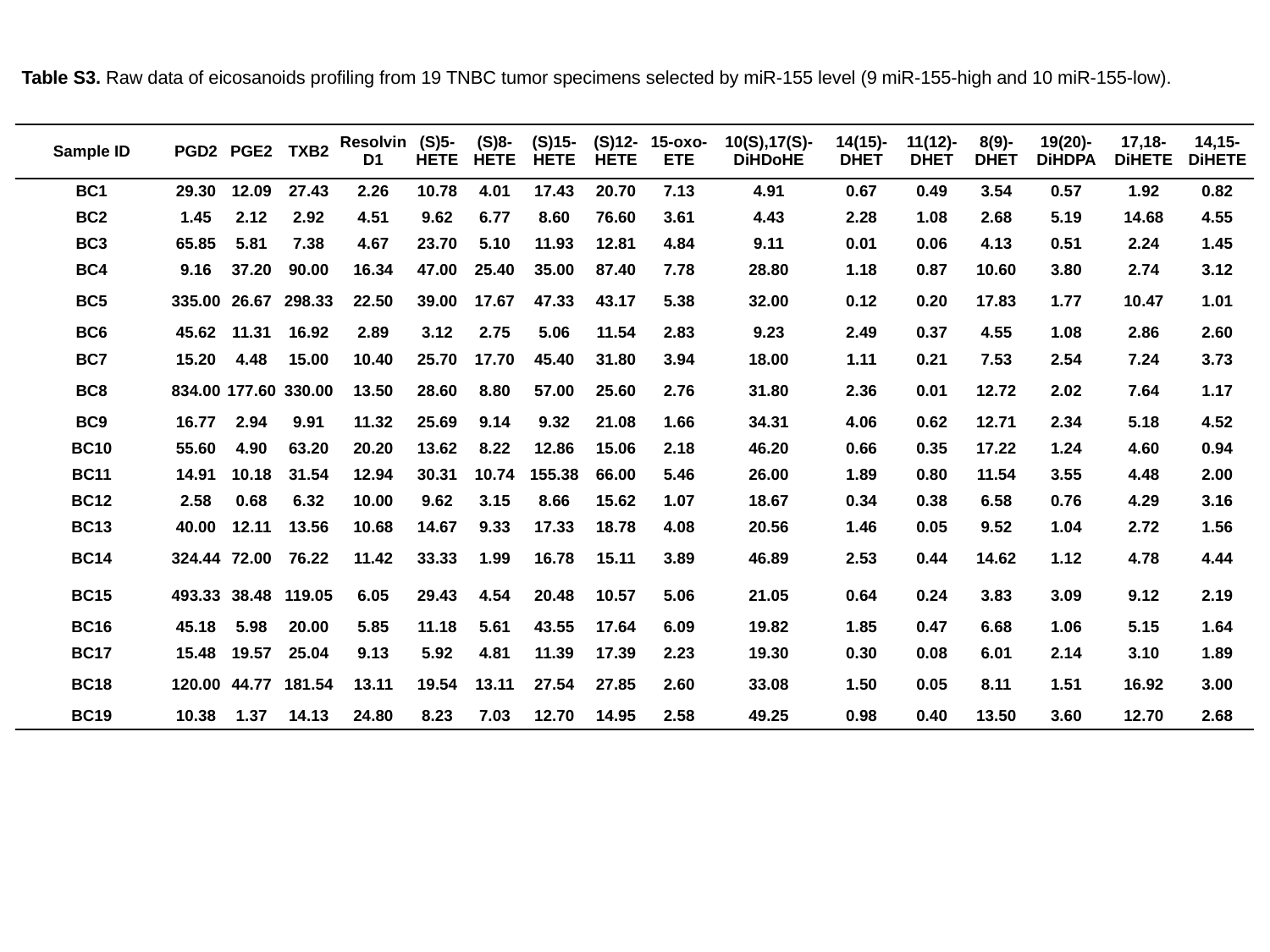

Table S3. Raw data of eicosanoids profiling from 19 TNBC tumor specimens selected by miR-155 level (9 miR-155-high and 10 miR-155-low).
| Sample ID | PGD2 | PGE2 | TXB2 | Resolvin D1 | (S)5-HETE | (S)8-HETE | (S)15-HETE | (S)12-HETE | 15-oxo-ETE | 10(S),17(S)-DiHDoHE | 14(15)-DHET | 11(12)-DHET | 8(9)-DHET | 19(20)-DiHDPA | 17,18-DiHETE | 14,15-DiHETE |
| --- | --- | --- | --- | --- | --- | --- | --- | --- | --- | --- | --- | --- | --- | --- | --- | --- |
| BC1 | 29.30 | 12.09 | 27.43 | 2.26 | 10.78 | 4.01 | 17.43 | 20.70 | 7.13 | 4.91 | 0.67 | 0.49 | 3.54 | 0.57 | 1.92 | 0.82 |
| BC2 | 1.45 | 2.12 | 2.92 | 4.51 | 9.62 | 6.77 | 8.60 | 76.60 | 3.61 | 4.43 | 2.28 | 1.08 | 2.68 | 5.19 | 14.68 | 4.55 |
| BC3 | 65.85 | 5.81 | 7.38 | 4.67 | 23.70 | 5.10 | 11.93 | 12.81 | 4.84 | 9.11 | 0.01 | 0.06 | 4.13 | 0.51 | 2.24 | 1.45 |
| BC4 | 9.16 | 37.20 | 90.00 | 16.34 | 47.00 | 25.40 | 35.00 | 87.40 | 7.78 | 28.80 | 1.18 | 0.87 | 10.60 | 3.80 | 2.74 | 3.12 |
| BC5 | 335.00 | 26.67 | 298.33 | 22.50 | 39.00 | 17.67 | 47.33 | 43.17 | 5.38 | 32.00 | 0.12 | 0.20 | 17.83 | 1.77 | 10.47 | 1.01 |
| BC6 | 45.62 | 11.31 | 16.92 | 2.89 | 3.12 | 2.75 | 5.06 | 11.54 | 2.83 | 9.23 | 2.49 | 0.37 | 4.55 | 1.08 | 2.86 | 2.60 |
| BC7 | 15.20 | 4.48 | 15.00 | 10.40 | 25.70 | 17.70 | 45.40 | 31.80 | 3.94 | 18.00 | 1.11 | 0.21 | 7.53 | 2.54 | 7.24 | 3.73 |
| BC8 | 834.00 | 177.60 | 330.00 | 13.50 | 28.60 | 8.80 | 57.00 | 25.60 | 2.76 | 31.80 | 2.36 | 0.01 | 12.72 | 2.02 | 7.64 | 1.17 |
| BC9 | 16.77 | 2.94 | 9.91 | 11.32 | 25.69 | 9.14 | 9.32 | 21.08 | 1.66 | 34.31 | 4.06 | 0.62 | 12.71 | 2.34 | 5.18 | 4.52 |
| BC10 | 55.60 | 4.90 | 63.20 | 20.20 | 13.62 | 8.22 | 12.86 | 15.06 | 2.18 | 46.20 | 0.66 | 0.35 | 17.22 | 1.24 | 4.60 | 0.94 |
| BC11 | 14.91 | 10.18 | 31.54 | 12.94 | 30.31 | 10.74 | 155.38 | 66.00 | 5.46 | 26.00 | 1.89 | 0.80 | 11.54 | 3.55 | 4.48 | 2.00 |
| BC12 | 2.58 | 0.68 | 6.32 | 10.00 | 9.62 | 3.15 | 8.66 | 15.62 | 1.07 | 18.67 | 0.34 | 0.38 | 6.58 | 0.76 | 4.29 | 3.16 |
| BC13 | 40.00 | 12.11 | 13.56 | 10.68 | 14.67 | 9.33 | 17.33 | 18.78 | 4.08 | 20.56 | 1.46 | 0.05 | 9.52 | 1.04 | 2.72 | 1.56 |
| BC14 | 324.44 | 72.00 | 76.22 | 11.42 | 33.33 | 1.99 | 16.78 | 15.11 | 3.89 | 46.89 | 2.53 | 0.44 | 14.62 | 1.12 | 4.78 | 4.44 |
| BC15 | 493.33 | 38.48 | 119.05 | 6.05 | 29.43 | 4.54 | 20.48 | 10.57 | 5.06 | 21.05 | 0.64 | 0.24 | 3.83 | 3.09 | 9.12 | 2.19 |
| BC16 | 45.18 | 5.98 | 20.00 | 5.85 | 11.18 | 5.61 | 43.55 | 17.64 | 6.09 | 19.82 | 1.85 | 0.47 | 6.68 | 1.06 | 5.15 | 1.64 |
| BC17 | 15.48 | 19.57 | 25.04 | 9.13 | 5.92 | 4.81 | 11.39 | 17.39 | 2.23 | 19.30 | 0.30 | 0.08 | 6.01 | 2.14 | 3.10 | 1.89 |
| BC18 | 120.00 | 44.77 | 181.54 | 13.11 | 19.54 | 13.11 | 27.54 | 27.85 | 2.60 | 33.08 | 1.50 | 0.05 | 8.11 | 1.51 | 16.92 | 3.00 |
| BC19 | 10.38 | 1.37 | 14.13 | 24.80 | 8.23 | 7.03 | 12.70 | 14.95 | 2.58 | 49.25 | 0.98 | 0.40 | 13.50 | 3.60 | 12.70 | 2.68 |

## Slide 14
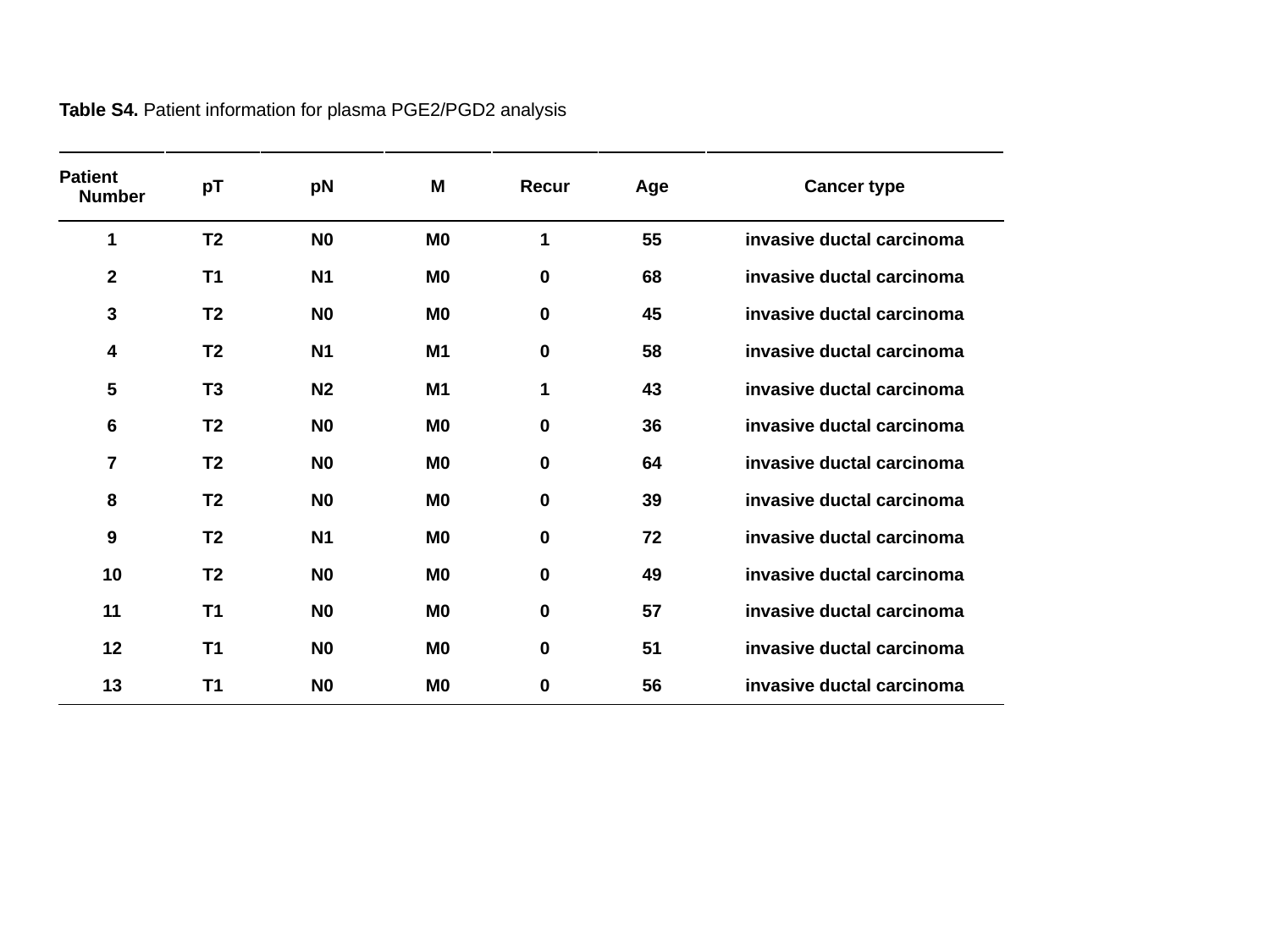

.
Table S4. Patient information for plasma PGE2/PGD2 analysis
| Patient Number | pT | pN | M | Recur | Age | Cancer type |
| --- | --- | --- | --- | --- | --- | --- |
| 1 | T2 | N0 | M0 | 1 | 55 | invasive ductal carcinoma |
| 2 | T1 | N1 | M0 | 0 | 68 | invasive ductal carcinoma |
| 3 | T2 | N0 | M0 | 0 | 45 | invasive ductal carcinoma |
| 4 | T2 | N1 | M1 | 0 | 58 | invasive ductal carcinoma |
| 5 | T3 | N2 | M1 | 1 | 43 | invasive ductal carcinoma |
| 6 | T2 | N0 | M0 | 0 | 36 | invasive ductal carcinoma |
| 7 | T2 | N0 | M0 | 0 | 64 | invasive ductal carcinoma |
| 8 | T2 | N0 | M0 | 0 | 39 | invasive ductal carcinoma |
| 9 | T2 | N1 | M0 | 0 | 72 | invasive ductal carcinoma |
| 10 | T2 | N0 | M0 | 0 | 49 | invasive ductal carcinoma |
| 11 | T1 | N0 | M0 | 0 | 57 | invasive ductal carcinoma |
| 12 | T1 | N0 | M0 | 0 | 51 | invasive ductal carcinoma |
| 13 | T1 | N0 | M0 | 0 | 56 | invasive ductal carcinoma |

## Slide 15
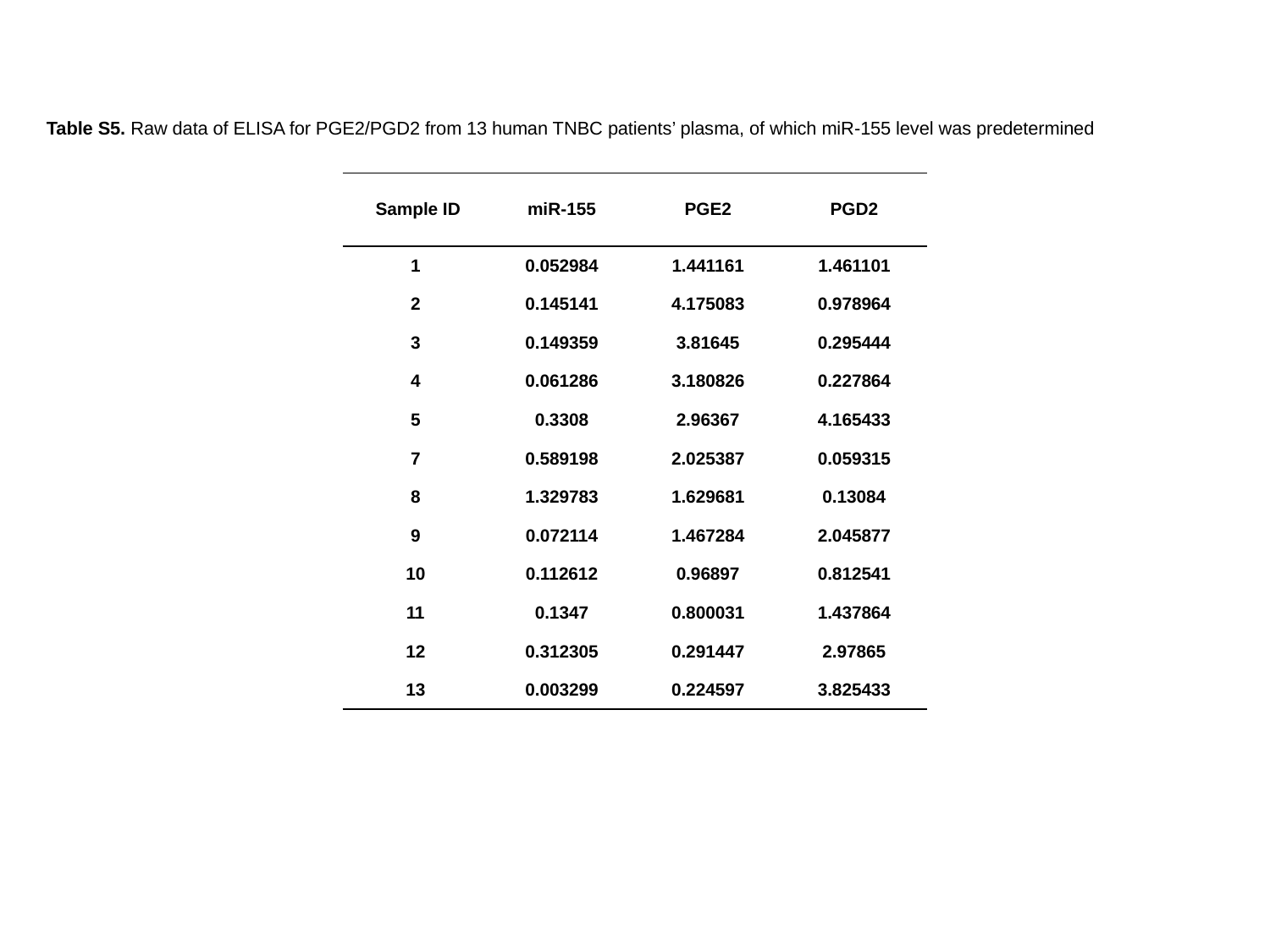

Table S5. Raw data of ELISA for PGE2/PGD2 from 13 human TNBC patients’ plasma, of which miR-155 level was predetermined
| Sample ID | miR-155 | PGE2 | PGD2 |
| --- | --- | --- | --- |
| 1 | 0.052984 | 1.441161 | 1.461101 |
| 2 | 0.145141 | 4.175083 | 0.978964 |
| 3 | 0.149359 | 3.81645 | 0.295444 |
| 4 | 0.061286 | 3.180826 | 0.227864 |
| 5 | 0.3308 | 2.96367 | 4.165433 |
| 7 | 0.589198 | 2.025387 | 0.059315 |
| 8 | 1.329783 | 1.629681 | 0.13084 |
| 9 | 0.072114 | 1.467284 | 2.045877 |
| 10 | 0.112612 | 0.96897 | 0.812541 |
| 11 | 0.1347 | 0.800031 | 1.437864 |
| 12 | 0.312305 | 0.291447 | 2.97865 |
| 13 | 0.003299 | 0.224597 | 3.825433 |

## Slide 16
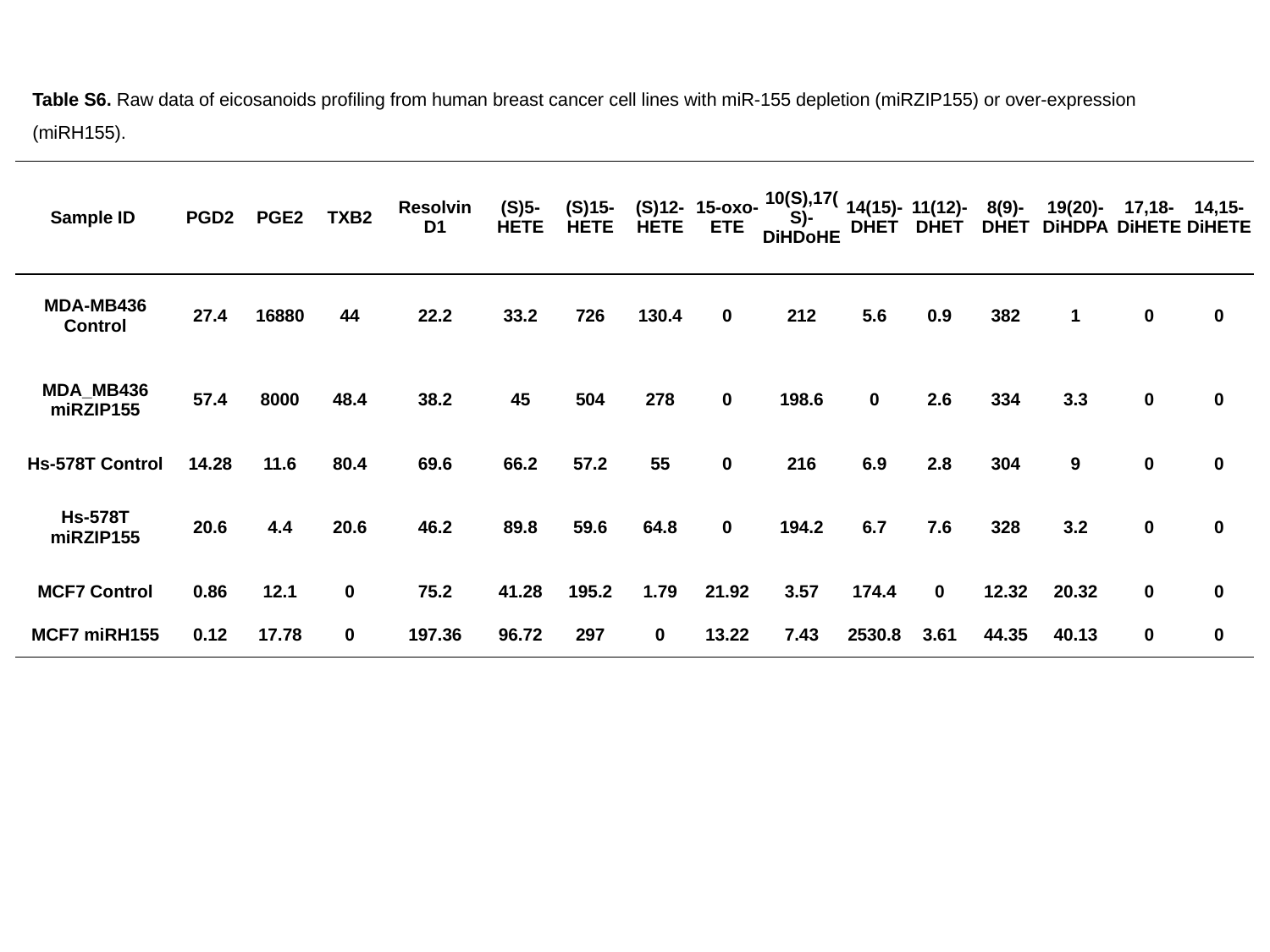

Table S6. Raw data of eicosanoids profiling from human breast cancer cell lines with miR-155 depletion (miRZIP155) or over-expression (miRH155).
| Sample ID | PGD2 | PGE2 | TXB2 | Resolvin D1 | (S)5-HETE | (S)15-HETE | (S)12-HETE | 15-oxo-ETE | 10(S),17(S)-DiHDoHE | 14(15)-DHET | 11(12)-DHET | 8(9)-DHET | 19(20)-DiHDPA | 17,18-DiHETE | 14,15-DiHETE |
| --- | --- | --- | --- | --- | --- | --- | --- | --- | --- | --- | --- | --- | --- | --- | --- |
| MDA-MB436 Control | 27.4 | 16880 | 44 | 22.2 | 33.2 | 726 | 130.4 | 0 | 212 | 5.6 | 0.9 | 382 | 1 | 0 | 0 |
| MDA\_MB436 miRZIP155 | 57.4 | 8000 | 48.4 | 38.2 | 45 | 504 | 278 | 0 | 198.6 | 0 | 2.6 | 334 | 3.3 | 0 | 0 |
| Hs-578T Control | 14.28 | 11.6 | 80.4 | 69.6 | 66.2 | 57.2 | 55 | 0 | 216 | 6.9 | 2.8 | 304 | 9 | 0 | 0 |
| Hs-578T miRZIP155 | 20.6 | 4.4 | 20.6 | 46.2 | 89.8 | 59.6 | 64.8 | 0 | 194.2 | 6.7 | 7.6 | 328 | 3.2 | 0 | 0 |
| MCF7 Control | 0.86 | 12.1 | 0 | 75.2 | 41.28 | 195.2 | 1.79 | 21.92 | 3.57 | 174.4 | 0 | 12.32 | 20.32 | 0 | 0 |
| MCF7 miRH155 | 0.12 | 17.78 | 0 | 197.36 | 96.72 | 297 | 0 | 13.22 | 7.43 | 2530.8 | 3.61 | 44.35 | 40.13 | 0 | 0 |

## Slide 17
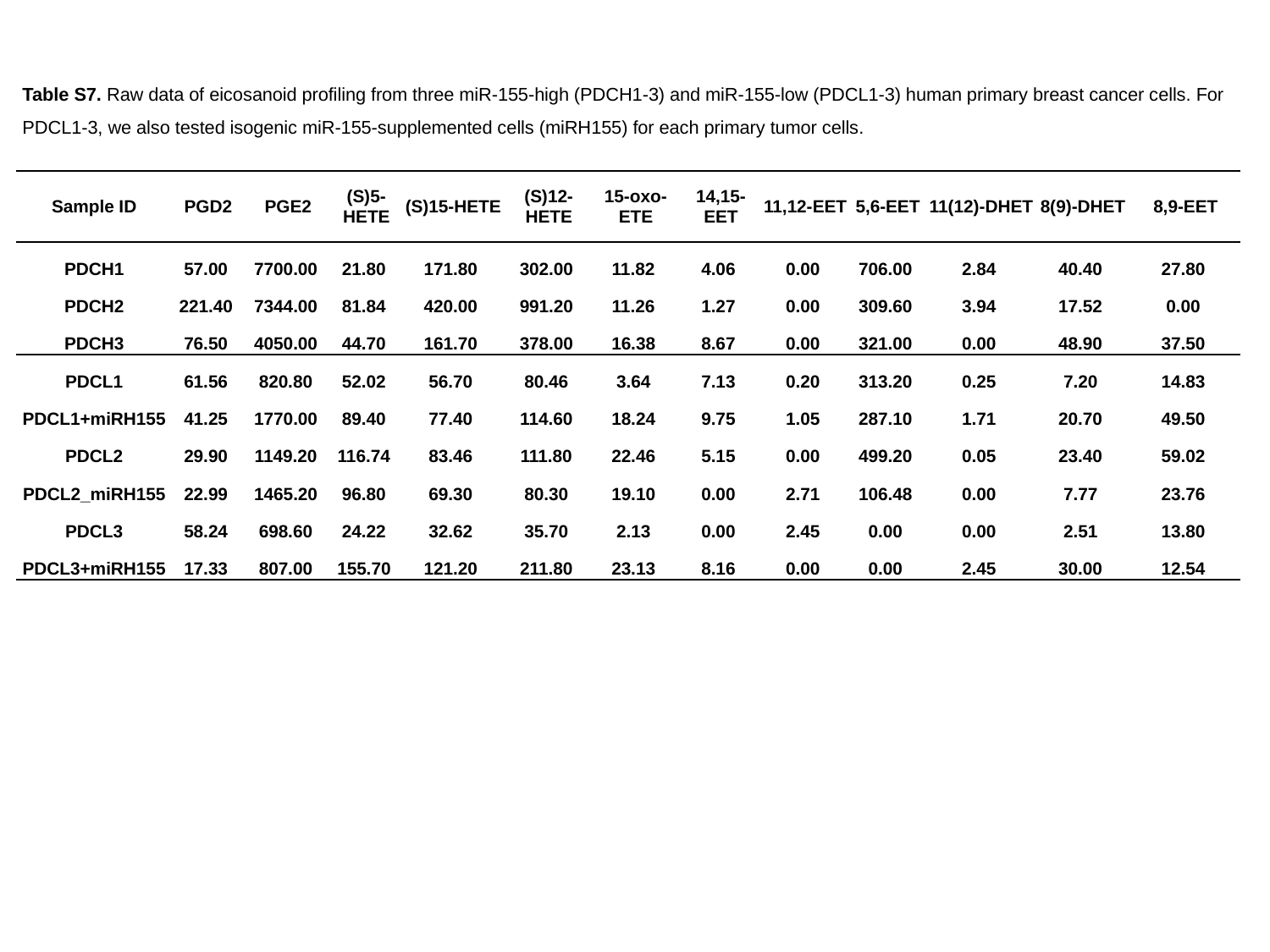

Table S7. Raw data of eicosanoid profiling from three miR-155-high (PDCH1-3) and miR-155-low (PDCL1-3) human primary breast cancer cells. For PDCL1-3, we also tested isogenic miR-155-supplemented cells (miRH155) for each primary tumor cells.
| Sample ID | PGD2 | PGE2 | (S)5-HETE | (S)15-HETE | (S)12-HETE | 15-oxo-ETE | 14,15-EET | 11,12-EET | 5,6-EET | 11(12)-DHET | 8(9)-DHET | 8,9-EET |
| --- | --- | --- | --- | --- | --- | --- | --- | --- | --- | --- | --- | --- |
| PDCH1 | 57.00 | 7700.00 | 21.80 | 171.80 | 302.00 | 11.82 | 4.06 | 0.00 | 706.00 | 2.84 | 40.40 | 27.80 |
| PDCH2 | 221.40 | 7344.00 | 81.84 | 420.00 | 991.20 | 11.26 | 1.27 | 0.00 | 309.60 | 3.94 | 17.52 | 0.00 |
| PDCH3 | 76.50 | 4050.00 | 44.70 | 161.70 | 378.00 | 16.38 | 8.67 | 0.00 | 321.00 | 0.00 | 48.90 | 37.50 |
| PDCL1 | 61.56 | 820.80 | 52.02 | 56.70 | 80.46 | 3.64 | 7.13 | 0.20 | 313.20 | 0.25 | 7.20 | 14.83 |
| PDCL1+miRH155 | 41.25 | 1770.00 | 89.40 | 77.40 | 114.60 | 18.24 | 9.75 | 1.05 | 287.10 | 1.71 | 20.70 | 49.50 |
| PDCL2 | 29.90 | 1149.20 | 116.74 | 83.46 | 111.80 | 22.46 | 5.15 | 0.00 | 499.20 | 0.05 | 23.40 | 59.02 |
| PDCL2\_miRH155 | 22.99 | 1465.20 | 96.80 | 69.30 | 80.30 | 19.10 | 0.00 | 2.71 | 106.48 | 0.00 | 7.77 | 23.76 |
| PDCL3 | 58.24 | 698.60 | 24.22 | 32.62 | 35.70 | 2.13 | 0.00 | 2.45 | 0.00 | 0.00 | 2.51 | 13.80 |
| PDCL3+miRH155 | 17.33 | 807.00 | 155.70 | 121.20 | 211.80 | 23.13 | 8.16 | 0.00 | 0.00 | 2.45 | 30.00 | 12.54 |

## Slide 18
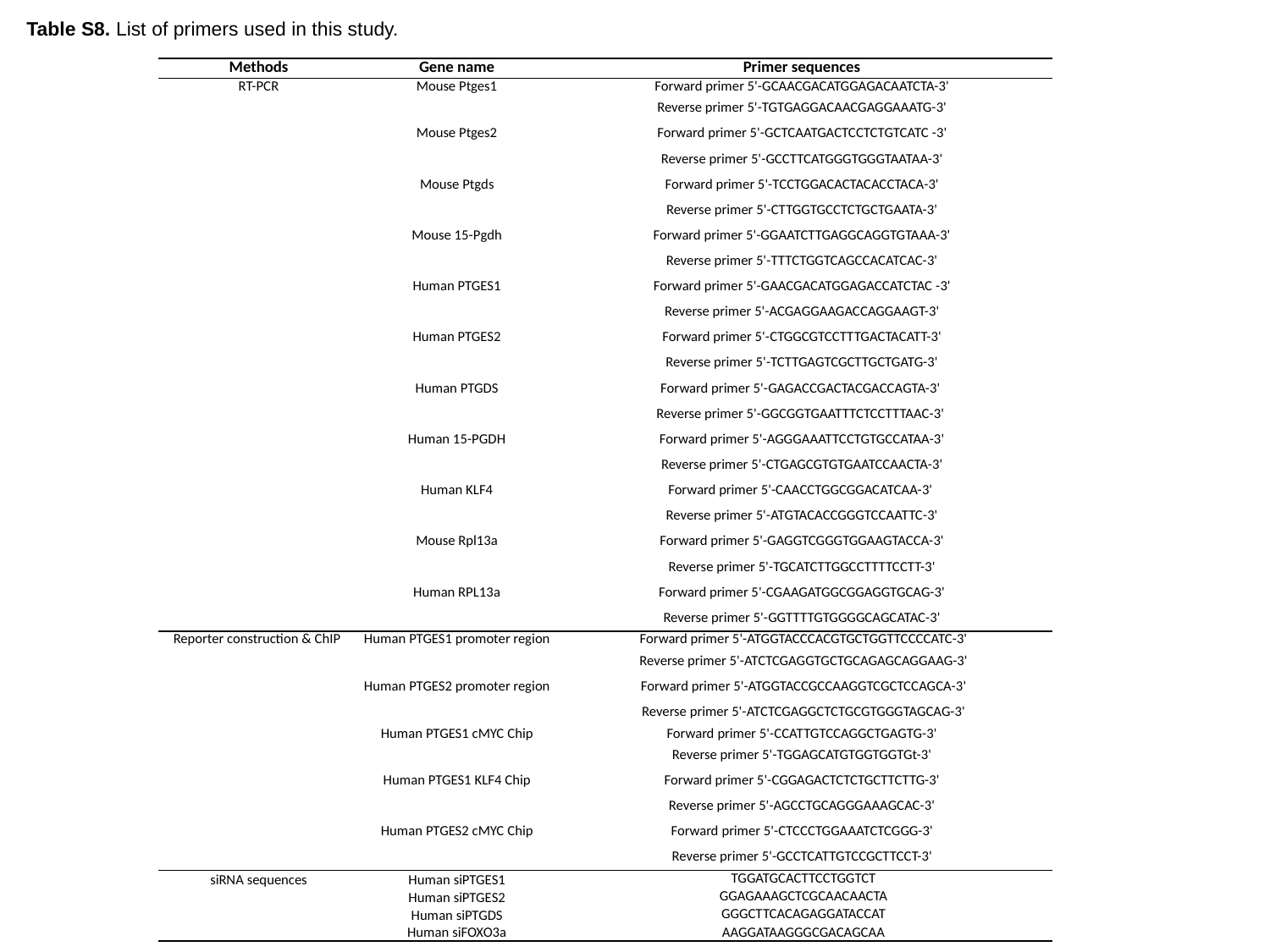

Table S8. List of primers used in this study.
| Methods | Gene name | Primer sequences |
| --- | --- | --- |
| RT-PCR | Mouse Ptges1 | Forward primer 5'-GCAACGACATGGAGACAATCTA-3' |
| | | Reverse primer 5'-TGTGAGGACAACGAGGAAATG-3' |
| | Mouse Ptges2 | Forward primer 5'-GCTCAATGACTCCTCTGTCATC -3' |
| | | Reverse primer 5'-GCCTTCATGGGTGGGTAATAA-3' |
| | Mouse Ptgds | Forward primer 5'-TCCTGGACACTACACCTACA-3' |
| | | Reverse primer 5'-CTTGGTGCCTCTGCTGAATA-3' |
| | Mouse 15-Pgdh | Forward primer 5'-GGAATCTTGAGGCAGGTGTAAA-3' |
| | | Reverse primer 5'-TTTCTGGTCAGCCACATCAC-3' |
| | Human PTGES1 | Forward primer 5'-GAACGACATGGAGACCATCTAC -3' |
| | | Reverse primer 5'-ACGAGGAAGACCAGGAAGT-3' |
| | Human PTGES2 | Forward primer 5'-CTGGCGTCCTTTGACTACATT-3' |
| | | Reverse primer 5'-TCTTGAGTCGCTTGCTGATG-3' |
| | Human PTGDS | Forward primer 5'-GAGACCGACTACGACCAGTA-3' |
| | | Reverse primer 5'-GGCGGTGAATTTCTCCTTTAAC-3' |
| | Human 15-PGDH | Forward primer 5'-AGGGAAATTCCTGTGCCATAA-3' |
| | | Reverse primer 5'-CTGAGCGTGTGAATCCAACTA-3' |
| | Human KLF4 | Forward primer 5'-CAACCTGGCGGACATCAA-3' |
| | | Reverse primer 5'-ATGTACACCGGGTCCAATTC-3' |
| | Mouse Rpl13a | Forward primer 5'-GAGGTCGGGTGGAAGTACCA-3' |
| | | Reverse primer 5'-TGCATCTTGGCCTTTTCCTT-3' |
| | Human RPL13a | Forward primer 5'-CGAAGATGGCGGAGGTGCAG-3' |
| | | Reverse primer 5'-GGTTTTGTGGGGCAGCATAC-3' |
| Reporter construction & ChIP | Human PTGES1 promoter region | Forward primer 5'-ATGGTACCCACGTGCTGGTTCCCCATC-3' |
| | | Reverse primer 5'-ATCTCGAGGTGCTGCAGAGCAGGAAG-3' |
| | Human PTGES2 promoter region | Forward primer 5'-ATGGTACCGCCAAGGTCGCTCCAGCA-3' |
| | | Reverse primer 5'-ATCTCGAGGCTCTGCGTGGGTAGCAG-3' |
| | Human PTGES1 cMYC Chip | Forward primer 5'-CCATTGTCCAGGCTGAGTG-3' |
| | | Reverse primer 5'-TGGAGCATGTGGTGGTGt-3' |
| | Human PTGES1 KLF4 Chip | Forward primer 5'-CGGAGACTCTCTGCTTCTTG-3' |
| | | Reverse primer 5'-AGCCTGCAGGGAAAGCAC-3' |
| | Human PTGES2 cMYC Chip | Forward primer 5'-CTCCCTGGAAATCTCGGG-3' |
| | | Reverse primer 5'-GCCTCATTGTCCGCTTCCT-3' |
| siRNA sequences | Human siPTGES1 | TGGATGCACTTCCTGGTCT |
| | Human siPTGES2 | GGAGAAAGCTCGCAACAACTA |
| | Human siPTGDS | GGGCTTCACAGAGGATACCAT |
| | Human siFOXO3a | AAGGATAAGGGCGACAGCAA |
